# Classification of High-Grade Serous Ovarian Carcinoma by Epithelial-to-Mesenchymal Transition Signature and Homologous Recombination Repair Genes

**DOI:** 10.3390/genes12071103

**Published:** 2021-07-20

**Authors:** Min-Hwan Sohn, Se Ik Kim, Jong-Yeon Shin, Hee Seung Kim, Hyun Hoon Chung, Jae-Weon Kim, Maria Lee, Jeong-Sun Seo

**Affiliations:** 1 Precision Medicine Center, Seoul National University Bundang Hospital, Seongnam-si 13605, Korea; phenomata@snu.ac.kr; 2 Precision Medicine Institute, Macrogen Inc., Seongnam-si 13605, Korea; jongyeon.anna@gmail.com; 3 Department of Biomedical Sciences, Seoul National University Graduate School, Seoul 03080, Korea; 4 Department of Obstetrics and Gynecology, Seoul National University College of Medicine, Seoul 03080, Korea; seikkim1@snu.ac.kr (S.I.K.); bboddi0311@snu.ac.kr (H.S.K.); chhkmj1@snu.ac.kr (H.H.C.); kjwksh@snu.ac.kr (J.-W.K.); 5 Department of Obstetrics and Gynecology, Seoul National University Hospital, Seoul 03080, Korea; 6 Gong-wu Genomic Medicine Institute, Seongnam-si 13605, Korea

**Keywords:** ovarian cancer, high-grade serous carcinoma, gene signature, epithelial-to-mesenchymal transition, homologous recombination repair

## Abstract

High-grade serous ovarian cancer (HGSOC) is one of the deadliest cancers that can occur in women. This study aimed to investigate the molecular characteristics of HGSOC through integrative analysis of multi-omics data. We used fresh-frozen, chemotherapy-naïve primary ovarian cancer tissues and matched blood samples of HGSOC patients and conducted next-generation whole-exome sequencing (WES) and RNA sequencing (RNA-seq). Genomic and transcriptomic profiles were comprehensively compared between patients with germline *BRCA1/2* mutations and others with wild-type *BRCA1/2*. HGSOC samples initially divided into two groups by the presence of germline *BRCA1/2* mutations showed mutually exclusive somatic mutation patterns, yet the implementation of high-dimensional analysis of RNA-seq and application of epithelial-to-mesenchymal (EMT) index onto the HGSOC samples revealed that they can be divided into two subtypes; homologous recombination repair (HRR)-activated type and mesenchymal type. Patients with mesenchymal HGSOC, characterized by the activation of the EMT transcriptional program, low genomic alteration and diverse cell-type compositions, exhibited significantly worse overall survival than did those with HRR-activated HGSOC (*p* = 0.002). In validation with The Cancer Genome Atlas (TCGA) HGSOC data, patients with a high EMT index (≥the median) showed significantly worse overall survival than did those with a low EMT index (<the median) (*p* = 0.030). In conclusion, through a comprehensive multi-omics approach towards our HGSOC cohorts, two distinctive types of HGSOC (HRR-activated and mesenchymal) were identified. Our novel EMT index seems to be a potential prognostic biomarker for HGSOC.

## 1. Introduction

Ovarian cancer, one of the deadliest gynecologic malignancies, is a global burden with an estimated 313,959 new cases and 207,252 cancer deaths each year [1]. The majority of ovarian cancers are epithelial ovarian cancers, and high-grade serous ovarian carcinoma (HGSOC) is the most prevalent histologic type [2]. In patients with HGSOC, germline or somatic mutations in *BRCA1* or *BRCA2* gene are frequently observed, and women harboring germline *BRCA1/2* mutations are at high risk of developing HGSOC [3].

The patients’ *BRAC1/2* mutational status is of high interest because several poly (adenosine diphosphate-ribose) polymerase (PARP) inhibitors are currently available for the treatment of primary and recurrent HGSOC, based on the phase 3 clinical trials, which have demonstrated the significant survival benefit brought by PARP inhibitors [4,5,6,7,8]. Beyond *BRCA1/2* genes, there is a need to discover other genetic mutations and altered gene expression programs that might be possible prognostic biomarkers or therapeutic targets.

One important feature of HGSOCs is that they are commonly diagnosed at an advanced stage, therefore showing high disease recurrence and mortality rates despite the primary treatment [9]. Researchers have noted epithelial-to-mesenchymal transition (EMT), a process referring to the conversion of an epithelial to a mesenchymal cell, as the mechanism for invasion and metastasis of ovarian cancer cells [10], as well as for achieving chemoresistance [11]. Interestingly, in breast cancer, loss of BRCA1 protein is associated with EMT [12]. However, such a relationship has been poorly investigated in ovarian cancer. Broadening the molecular understanding of HGSOC and elucidating the underlying mechanisms for EMT in terms of *BRCA1/2* gene alterations is expected to open a new horizon in the treatment of HGSOC [13].

In this regard, we carried out next-generation whole-exome sequencing (WES) and RNA sequencing (RNA-seq) to find the causal variants that bring about HGSOC in terms of homologous recombination repair (HRR) genes and EMT.

## 2. Materials and Methods

### 2.1. Study Population

Inclusion criteria for the study population were as follows: (1) diagnosed with HGSOC between January 2013 and December 2016; (2) having undergone primary debulking surgery; (3) having donated their blood samples, obtained one day before surgery, and fresh-frozen primary ovarian cancer tissues, obtained at the time of surgery, for scientific purposes after providing written informed consent; and (4) having an identifiable germline *BRCA1/2* mutational status. In addition, patients were excluded if (1) they had any malignancy other than HGSOC; (2) received neoadjuvant chemotherapy; or (3) had insufficient clinical data or were lost to follow-up.

Among patients who met these criteria, we further selected patients referring to their germline *BRCA1/2* genetic test results as follows: (1) five patients harboring germline deleterious *BRCA1* mutations and wild-type *BRCA2* (g*BRCA1*mut); (2) five patients harboring germline deleterious *BRCA2* mutations and wild-type *BRCA1* (g*BRCA2*mut); and (3) 10 patients with wild-type *BRCA1/2* genes (g*BRCA1/2*wt). Details of the germline *BRCA1/2* gene testing methods at our institution were described in a previous study [14].

We collected the patients’ baseline clinicopathologic characteristics, such as age at diagnosis, International Federation of Gynecology and Obstetrics (FIGO) stage, initial serum CA-125 levels, and residual tumor size after surgery. In terms of survival outcomes, progression-free survival (PFS) was defined as the time interval between the date of diagnosis to the date of disease progression, while overall survival (OS) was defined as the time interval between the date of diagnosis to the date of cancer-related death or last visit.

### 2.2. Whole-Exome Sequencing, RNA Sequencing, and Data Analysis

The fresh-frozen, primary ovarian cancer tissues and blood samples of 20 patients were retrieved from Seoul National University Hospital Human Biobank. One expert gynecologic pathologist (Cheol Lee) in Seoul National University Hospital reviewed and confirmed all the HGSOC cases in our study population according to the World Health Organization Classification of Tumors, 5th edition. Detailed methods for WES on the tumor tissues and matched blood samples, RNA-seq on the tumor tissues, and their analysis are presented in Supplementary Methods. The sequencing coverage and quality metrics of WES and RNA-seq are provided in Tables S1 and S2.

### 2.3. Transcription Factor Enrichment Analysis

Adding to the differentially expressed gene (DEG) analysis, principal component analysis (PCA), K-means clustering, and unsupervised hierarchical clustering (HC), we performed transcription factor enrichment analysis (TFEA) for a particular set of genes by using ChIP-X Enrichment Analysis version 3 [15]. Particularly, we used a complete list of transcription factors (TFs) and their target gene-set libraries from ARCHS4 [16], which is a compendium of publicly available, processed RNA-seq data (https://maayanlab.cloud/chea3/assets/tflibs/ARCHS4_Coexpression.gmt, accessed on 14 April 2021). We only used the top 10 enriched TFs with false discovery rate <0.05 for subsequent analyses.

### 2.4. Caculation of EMT Index

To analyze RNA-seq data in relation to EMT, we manually coined an index, the “EMT index”. Specifically, the EMT index was calculated for each sample based on the geometric mean of transcripts per million (TPM) values for five core EMT-TFs (*TWIST1*, *SNAI1*, *SNAI2*, *ZEB1*, and *ZEB2*) and 33 EMT-related TFs (*KLF4*, *GSC*, *TCF7L2*, *ALX1*, *GATA6*, *RUNX2*, *TCF3*, *SOX4*, *FOXC2*, *NFKB1*, *KLF2*, *KLF6*, *TBX3*, *TCF4*, *PRRX1*, *HOXB7*, *JUN*, *FOS*, *TAZ*, *TGIF1*, *ATF1*, *ERG*, *ETS1*, *ID1*, *TEAD1*, *YAP1*, *NFYA*, *KLF8*, *SOX9*, *SIX1*, *TBXT*, *GATA4*, and *TWIST2*) according to the consensus statement on EMT led by the EMT International Association (TEMTIA) [17].

### 2.5. Identification of Co-Expressed Gene Modules and Interaction Networks

To identify gene co-expression modules and interaction networks from RNA-seq data, we used CEMiTool [18] version 1.14.0. In total, 19,023 genes, upon which was applied variance-stabilizing transformation (vst) implemented in DESeq2 [19], were used as inputs and samples were divided into two pre-annotated clusters by K-means clustering, namely, cluster A and cluster B, with the following settings: corr_method = “spearman”, network type = “signed”, tom_type = “signed”, rank_method = “mean”, gsea_max_size = 2000. Calculated modules were considered significant only if the absolute value of normalized enrichment scores (NES) for both cluster A and cluster B was above 4 and with a Benjamini–Hochberg adjusted *p* value < 0.0001. For the input-constructing interaction network of each co-expressed gene module, we retrieved TFs target gene-set libraries from ARCHS4 [16] as a Gene Matrix Transposed (gmt) file format with a minor modification, putting TF genes and their target genes in the first column and the second column, respectively (https://github.com/ryansohny/HGSOC/blob/main/RNA-seq/ARCHS4_Coexpression_interaction.csv). Then, we performed overrepresentation analysis implemented in CEMiTool using HALLMARK gene sets from the Molecular Signature Database (MSigDB) [20].

### 2.6. Cell-Type Enrichment Analysis

To further validate our findings regarding classification of our samples into two groups based on their genomic and transcriptomic profiles, we performed cell-type enrichment analysis from gene expression data. An expression profile of samples was uploaded to XCell [21] web interface with default parameters using “xCell (N = 64)” gene signature.

### 2.7. Analysis of TCGA Data

We downloaded The Cancer Genome Atlas (TCGA) RNA-seq data of 376 HGSOC samples and corresponding clinicopathological profiles from the National Cancer Institute Genomic Data Commons Data Portal (https://portal.gdc.cancer.gov/, accessed on 22 February 2018) and cBioPortal for Cancer Genomics (https://www.cbioportal.org, accessed on 22 February 2018) website. TPM values were calculated by dividing each gene’s fragments per kilobase per million (FPKM) value with the sum of FPKM of that particular sample. To divide the TCGA cohort in terms of EMT index, the median value of the EMT indices of all samples was used; samples having a higher EMT index than the median value (11.999) were classified as EMT-high, while the remainders were classified as EMT-low.

### 2.8. Statistical Analysis

Differences in baseline characteristics and genomic or transcriptomic profiles between two groups (g*BRCA1*mut and g*BRCA1/2*wt) or among three (g*BRCA1*mut, g*BRCA2*mut, and g*BRCA1/2*wt) were assessed: Pearson’s chi-square or Fisher’s exact tests were used for categorical variables, while Student’s t-, Mann–Whitney U, ANOVA, or Kruskal–Wallis tests were used for continuous variables. Tukey’s HSD was used for multiple comparisons. Pearson correlation coefficients were calculated between patient characteristics and somatically mutated genes. Survival outcomes were compared using Kaplan–Meier analysis with log-rank test. R statistical software version 4.0.2 (R Foundation for Statistical Computing, Vienna, Austria) was used for the statistical analyses. *P* values < 0.05 were considered statistically significant unless otherwise noted.

## 3. Results

### 3.1. Characteristics and Survival Outcomes of Patients with HGSOC

**Figure 1:**
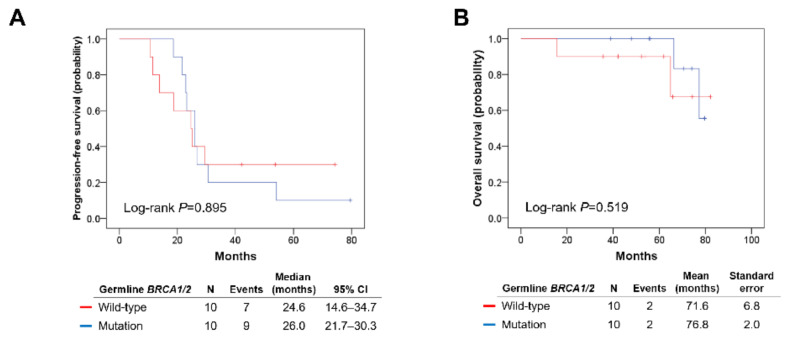
Comparisons of survival outcomes between germline *BRCA1/2* mutation and wild-type groups. (**A**) Progression-free survival. (**B**) Overall survival.

Between the g*BRCA1/2*mut and g*BRCA1/2*wt groups, no differences were observed in baseline clinicopathologic characteristics (Table 1). None of the study population received PARP inhibitors at their primary treatment, whereas three patients in the g*BRCA1/2*mut group received PARP inhibitor maintenance therapy to treat relapsed disease. A median observation period was 63.4 months. The two groups showed a similar PFS (median, 26.0 vs. 24.6 months; *p* = 0.895) and OS (mean, 76.8 vs. 71.6 months; *p* = 0.519; Figure 1A,B).

### 3.2. Genomic Profiling of HGSOC

**Figure 2:**
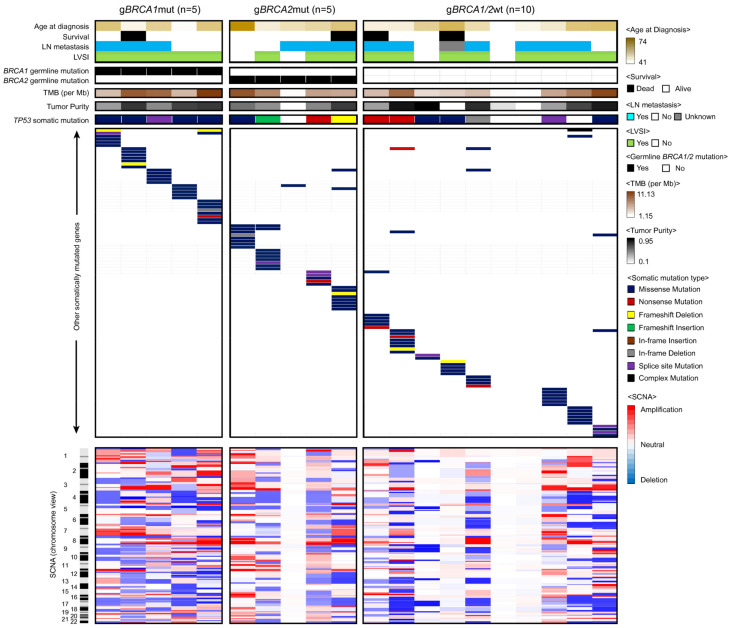
Genomic mutational characterization of 20 HGSOC samples. The distribution of somatic mutations among three categories of samples. Each column displayed here represents an individual case. LN, LVSI, TMB, and SCNA stand for lymph node, lymphovascular space invasion, tumor mutational burden, and somatic copy number alteration, respectively.

WES of 20 blood samples revealed the same germline *BRCA1/2* mutations as those identified by our in-house gene testing (Figure S1, Table S3). In detail, samples from the g*BRCA1*mut group had a frameshift insertion (g*BRCA1*mut_1), a frameshift deletion (g*BRCA1*mut_3, gBRCA1mut_4), and a stop-gain SNV (g*BRCA1*mut_2) in the *BRCA1* gene, which were all heterozygous, and a hemizygous deletion of exon 1 through 14 of the *BRCA1* gene (g*BRCA1*mut_5). All samples from the g*BRCA2*mut group had the frameshift deletion of a single *BRCA2* gene in five different sites (g*BRCA2*mut_1 to 5). Next, we investigated somatic mutations and putative drivers of HGSOC progression from tumor–normal pairs (Figure 2). Interestingly, we observed a mutually exclusive variants pattern with few co-occurring somatic single nucleotide variants (SNVs) and indels across our samples, except for the *TP53* mutation (pairwise Fisher’s exact test *p* > 0.05). The lack of *TP53* somatic mutations in some of our samples, which is rare in HGSOC, might originate from their low tumor purity. In particular, two g*BRCA1/2*wt samples lacked any apparent driver mutations of SNVs or indels. Tumor mutational burden (TMB) was assessed for each sample, but no significant difference was detected among the g*BRCA1*mut, g*BRCA2*mut, and g*BRCA1/2*wt groups (one-way ANOVA test *p* = 0.313) (Figure S2). In terms of somatic copy number alterations (SCNAs), we observed amplification of genes, such as *CSF3R*, *LCK*, *MPL*, *MUTYH*, *SFPQ*, *STIL*, and *TAL1*, and loss of genes, such as *GNA11*, *MLLT1*, *MAP2K2*, and *SH3GL1* (Figure S3).

### 3.3. Transcriptomic Profiling of HGSOC in Terms of HRR and EMT

**Figure 3:**
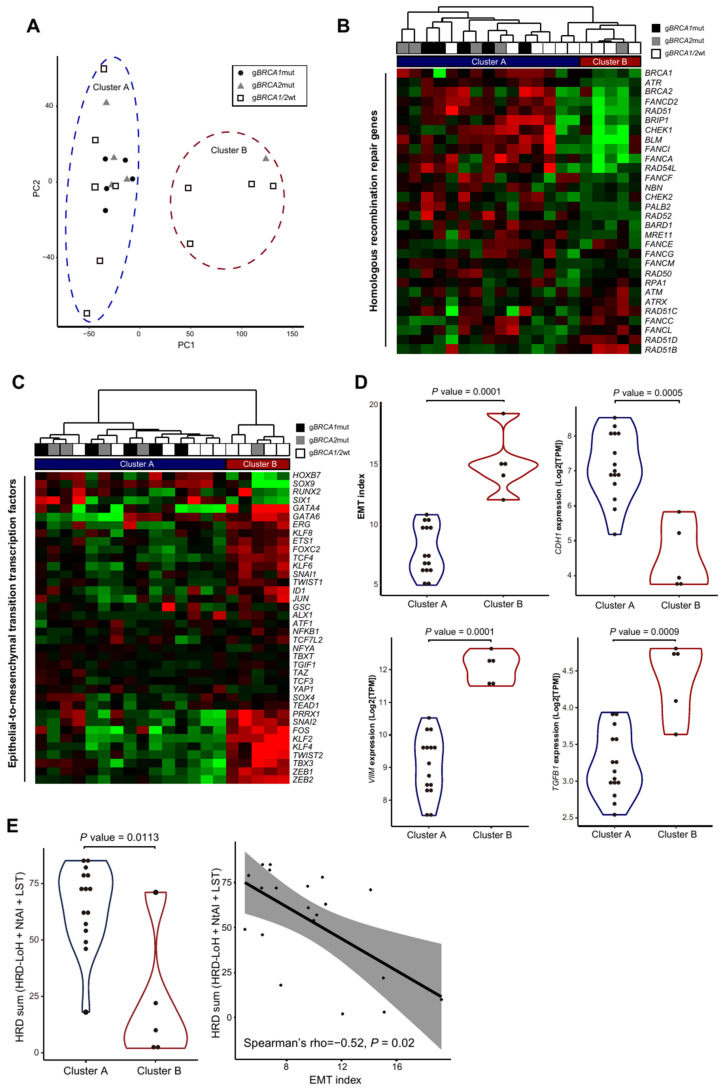
Two distinctive patterns of molecular subtype identified through RNA-seq data analysis. (**A**) Transcriptional landscape of HGSOC samples through principal component analysis. Samples are represented by different shapes and colors by their origin and grouped according to K-means clustering with k = 2 (cluster A and cluster B). (**B**) Hierarchical clustering of samples represents the expression profile of 30 HRR genes. (**C**) Hierarchical clustering of samples with the expression profile of 38 EMT-TFs reproduced the result from the PCA analysis. (**D**) Violin plots showing difference in EMT index and gene expressions of *CDH1*, *VIM*, and *TGFB1* between cluster A and cluster B. Each *p* value was calculated via Mann–Whitney U test. (**E**) A violin plot-view of HRD score distribution between cluster A and cluster B (left), and relationship between EMT-index and HRD sum scores (right). HRD scores between cluster A and cluster B were compared using Mann–Whitney U test. Statistical dependence between EMT index and HRD scores were computed through Spearman’s rank correlation coefficients. LoH, NtAI, and LST stand for loss of heterozygosity, number of telomeric allelic imbalances, and large-scale transition, respectively.

Based on the RNA-seq data from 20 HGSOC samples, we conducted PCA to cluster the samples on the basis of the top 5000 variable genes out of 19,023 genes, and observed highly similar transcriptomic profiles between the g*BRCA1*mut and g*BRCA2*mut groups (Figure 3A). Six out of 10 samples in the g*BRCA1/2*wt group were clustered into “cluster A” together with the g*BRCA1*mut and g*BRCA2*mut groups, with the exception of one g*BRCA2*mut sample. Meanwhile, the remaining four samples in the g*BRCA1/2*wt group and the g*BRCA2*mut sample were segregated into “cluster B” (Figure 3A). To determine the causal or regulatory variants for clusters A and B, we first performed TFEA for genes exhibiting a negative correlation (r < −0.9, *n* = 60) with the principal component (PC1) and that were upregulated in cluster A rather than in cluster B (Table S4). The most significantly enriched TF gene was *GRHL2*, known as an EMT suppressor in various cancers (Table S5).

Next, considering that cluster A included most samples of the g*BRCA1/2*mut group, we investigated transcriptomic aberration of the HRR genes (Table S6). Unsupervised hierarchical clustering of 30 HRR genes recapitulated the PCA result, and 18 out of 30 HRR genes (e.g., *ATR*, *FANCA*, and *FANCD2*) were significantly upregulated in cluster A rather than in cluster B (Figure 3B). The activation of HRR pathways might be explained by a genetic compensation for the dysfunction of *BRCA1* or *BRCA2* in the g*BRCA1/2*mut group, which accounts for a large part of cluster A. Furthermore, six samples from the g*BRCA1/2*wt group that fell into cluster A had several somatic alterations in HRR genes: missense mutations in *BRCA1*, *ATRX*, and *ATR*, copy number loss of *BRCA2*, *FANCC*, *FANCG*, and *RAD50*, and copy number gain of *RAD51B* and *RAD54L* (Figure S4). Then, in order to find specific TFs regulating the expression of HRR genes, we again conducted TFEA for the 18 upregulated HRR genes and discovered that *E2F8*, *E2F2*, *E2F3*, *PRDM9*, *CENPA*, and *TGIF* were the core regulators or components of the gene networks overexpressed in cluster A (Table S7).

Focusing on genes upregulated in cluster B compared to their expression in cluster A, we also performed TFEA for genes exhibiting a positive correlation (r > 0.9, *n* = 180) with PC1 (Table S3). Interestingly, among the enriched TFs (Table S8), *TCF21*, *TWIST2*, *MEOX2*, *OSR1*, *PRRX1*, *PRRX2*, and *TWIST1* were associated with EMT [22]. Investigation of the RNA expression of these TFs indicated that most of them were upregulated in cluster B rather than in cluster A (Figure S5).

Analyzing RNA-seq data in relation to EMT, we calculated the EMT index (Table S9). Unsupervised hierarchical clustering of samples with these 38 TFs accurately separated 20 HGSOC tissue samples into clusters A and B (Figure 3C). Between the two clusters, the EMT index was significantly different (*p* = 0.001; Figure 3D, top left).

In addition to the 38 genes used to calculate the EMT index, *CDH1* (E-cadherin), known to be highly expressed in epithelial tissue and downregulated in mesenchymal tissue [17], was downregulated in cluster B (Figure 3D, top right). In contrast, *VIM* (vimentin), another key indicator of EMT highly expressed in mesenchymal rather than in epithelial tissue [23], was upregulated in cluster B (Figure 3D, bottom left). In addition, *TGFB1* (TGFβ), known as a key accelerator of EMT [24], was also upregulated in cluster B (Figure 3D, bottom right).

Interestingly, homologous recombination deficiency (HRD) score [25], a genomic scar estimate combining three measures (loss of heterozygosity, telomeric allelic imbalance, and large-scale state transitions) was higher in cluster A, compared to that of cluster B (Figure 3E, left, Figure S6). Moreover, EMT index was found to be negatively correlated with the genomic scar estimate (Figure 3E, right).

**Figure 4:**
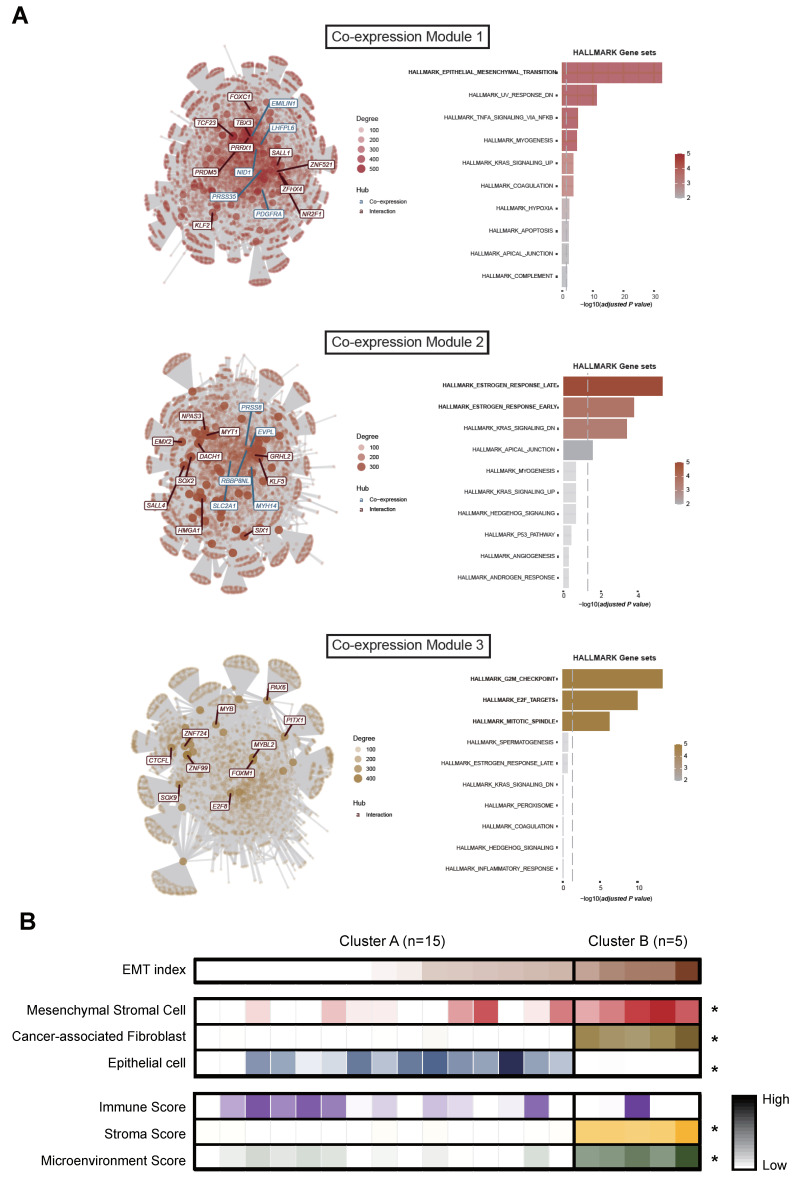
Co-expression gene module identification and cell-type enrichment. (**A**) Interaction network of identified gene modules and over representation analysis using HALLMARK gene sets. (**B**) EMT index and cell-type enrichment analysis results across 20 HGSOC samples divided by cluster A and cluster B and by order of increasing EMT-index. * Mann–Whitney U test *p* < 0.05 between cluster A and cluster B.

To dissect variation in the transcriptional network of our samples and further validate the transcriptional nature of two groups, cluster A and cluster B, we performed gene co-expression network analysis [18]. With this approach, we were able to identify one module (Co-expression Module 1) enriched in samples from cluster B, and two modules (Co-expression Modules 2 and 3) enriched in samples from cluster A (Figure 4A and Figure S7). Co-expression Module 1 had EMT-TFs (e.g., *KLF2* and *PRRX1*) as interaction hub genes, consistent with the finding that EMT gene signature was enriched in cluster B. Co-expression Modules 2 and 3 were characterized by distinctive hub genes such as *SLC2A1*, which is known to be regulated by estrogens [26], and *MYBL2*, a core regulator of cellular differentiation [27], was among the main components of the complex network of gene expression in cluster A.

Meanwhile, we found a negative correlation between PC1 and tumor purity, derived from WES data (r = −0.84, *p* < 0.001; Figure S8, Table S10), consistent with the finding that mesenchymal-type ovarian cancers tend to have lower tumor purity than do other types [28,29]. Using the gene expression data, we also conducted cell-type enrichment analysis [21]: the mesenchymal stromal cell, the intra-tumoral cancer-associated fibroblast (CAF), and epithelial cell signature were investigated (Figure 4B). Samples in cluster B were enriched in mesenchymal stromal cells and CAFs compared to samples in cluster A enriched in epithelial cells. Consistently, we also observed that two CAF marker genes, *DCN* and *PDPN*, were significantly upregulated in cluster B compared to their expression in cluster A (Figure S9).

Taken together, we could classify 20 HGSOC tissue samples into two categories: (1) HRR-activated HGSOC (cluster A) and (2) mesenchymal HGSOC (cluster B).

### 3.4. EMT Index and Survival Outcomes

We performed survival analysis between patients with mesenchymal HGSOC (*n* = 5) and those with HRR-activated HGSOC (*n* = 15). While the two groups showed similar PFS (*p* = 0.708), patients with mesenchymal HGSOC exhibited significantly worse OS than those with HRR-activated HGSOC (*p* = 0.002) (Figure S10).

**Figure 5:**
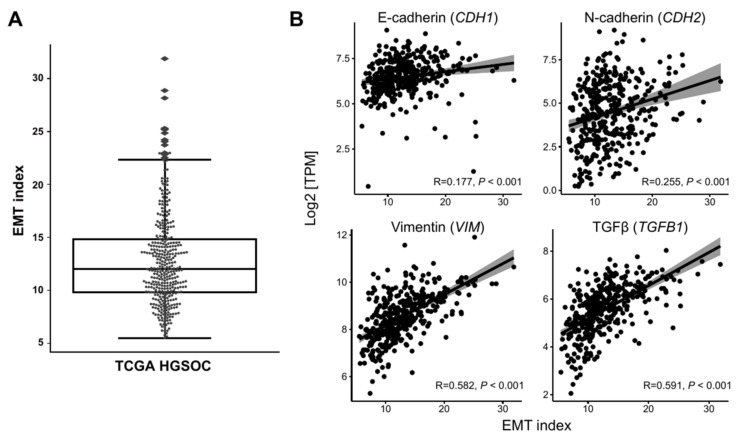

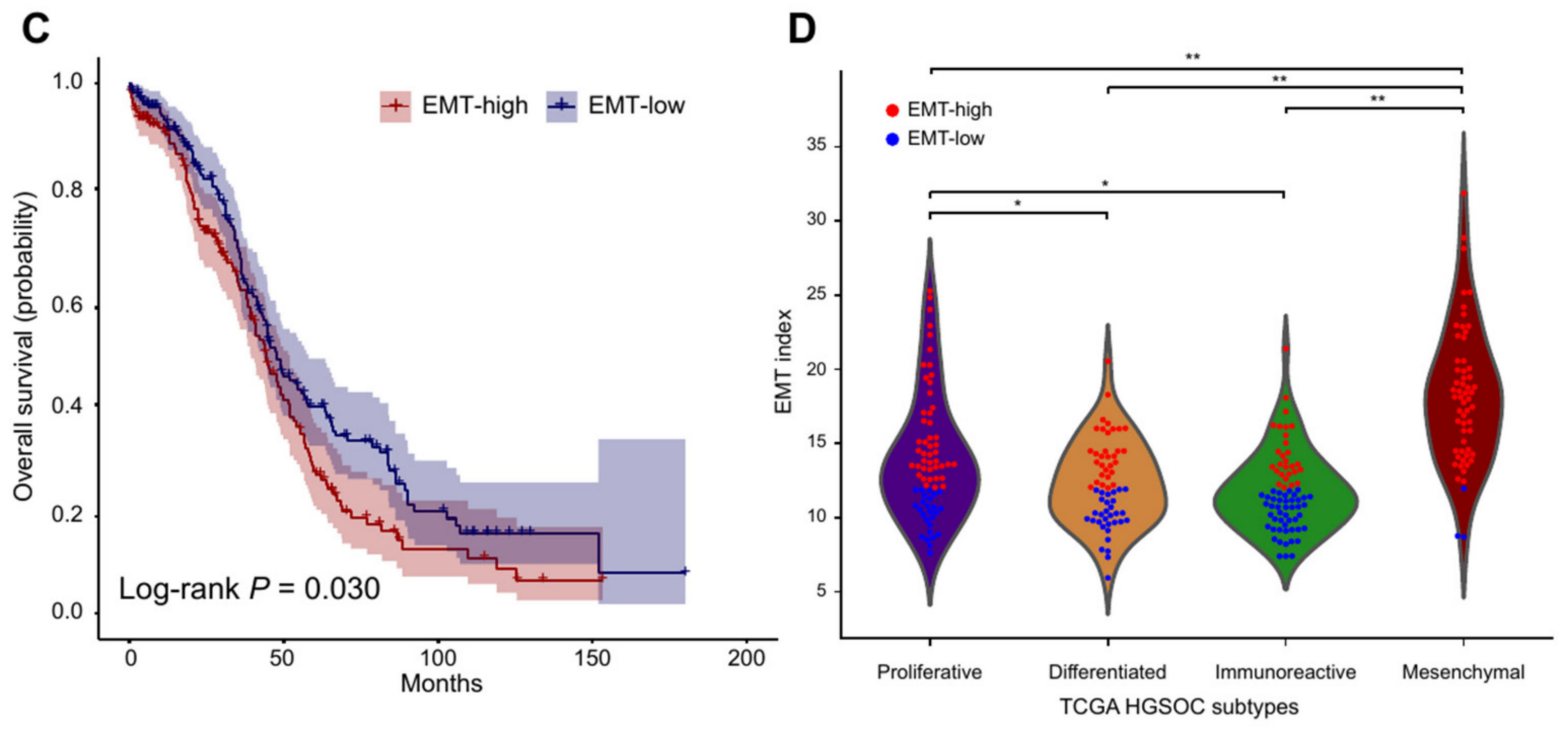
Application of the EMT index to TCGA HGSOC data. (**A**) Distribution of EMT index of TCGA HGSOC displayed on a box plot. (**B**) Scatter plots illustrating relationship between the EMT index and EMT-related gene expression in the cohort. Each dot represents each sample analyzed, and red lines are a linear trend representation of the scatter plots. (**C**) Kaplan–Meier plot depicting overall survival of TCGA HGSOC samples falling into EMT-high (red) and -low (blue) groups. (**D**) EMT index for four TCGA subtypes was compared and the mesenchymal subtype exhibited the highest EMT index (one-way ANOVA test *p* < 0.001; Tukey’s HSD adjusted *p* < 0.005 ** and < 0.05 *). Red dots and blue dots inside the violin plots represent EMT-high and -low samples, respectively.

Next, we investigated the reproducibility of our study findings using TCGA HGSOC data [30]. Processing 379 RNA-seq samples, we calculated each sample’s EMT index (Figure 5A) and examined its correlation with known EMT markers (Figure 5B). Although the expression of *CDH1*, which was expected to be decreased with the increasing EMT index, had a weak positive correlation with the EMT index (r = 0.177, *p* < 0.001), its presence in EMT-high samples might indicate epithelial/mesenchymal intermediate states or reflect transient activation and repression of the EMT program [31,32]. *CDH2*, encoding N-cadherin and serving as an indicator of EMT [33], was positively correlated with the EMT index (r = 0.255, *p* < 0.001), suggesting the possibly increased mesenchymal population within the EMT-high samples. *VIM* and *TGFB1* also increased with the rise in the EMT index (r = 0.582, *p* < 0.001; and r = 0.591, *p* < 0.001, respectively).

Then, we analyzed the survival outcomes by the level of EMT index in TCGA HGSOC samples for which survival data were available (*n* = 374) (Figure 5C). The OS of patients whose samples had a high EMT index (≥the median, *n* = 187) was significantly worse than that of patients whose samples had a low EMT index (<the median, *n* = 187) (median, 44.0 vs. 47.4 months; *p* = 0.030). Checking how the EMT-high and -low groups were distributed in the four subtypes of TCGA HGSOC (Figure 5D), we observed that the EMT-high samples were mostly enriched in the mesenchymal subtype (Chi-square test *p* < 0.001; Benjamini–Hochberg corrected *p* < 0.001 for all pairwise Fisher’s Exact test between mesenchymal and others). Moreover, among the four subtypes of TCGA HGSOC, the mesenchymal subtype exhibited the highest level of EMT index (one-way ANOVA test *p* < 0.001; adjusted *p* < 0.05 for all Tukey’s HSD).

## 4. Discussion

In this study, we investigated the molecular characteristics of HGSOC through an integrative analysis of genomic and transcriptomic data obtained from chemotherapy-naïve primary HGSOC tissues. Consequently, we could simplify the molecular classification of HGSOC to HRR-activated and mesenchymal types. The prognostic value of the EMT index was also validated using TCGA HGSOC data. Our study results demonstrate that the EMT index would be a potential prognostic biomarker for HGSOC.

Of two distinctive types of HGSOC, HRR-activated HGSOC was characterized by a malfunction of the HRR program caused by deficient *BRCA1/2* or HRR genes and the transcriptomic aberration of other HRR genes. Furthermore, we revealed that genes regulating or co-expressed with HRR genes are members of the E2F family (*E2F8*, *E2F2*, and *E2F3*), known as cell cycle regulators [34]; *PRDM9*, related to the process of meiosis and responsible for directing the positions of HRR [35]; *CENPA*, involved in accurate chromosome segregation [36]; and *TGIF*, reported to be over-expressed among ovarian cancer cell lines [37].

The other type, mesenchymal HGSOC, was characterized by low genomic alteration, transcriptional activation of EMT-TFs, decreased epithelial cell marker expression, increased mesenchymal cell marker expression, and diverse cell type composition. Regarding activation of EMT-TFs, a previous study in colorectal cancer reported that ZEB1, one of the core EMT-TFs, was activated through the β-catenin/TCF4 complex [38]. Similarly, we also observed upregulation of both β-catenin and TCF4 and of their target ZEB1 in mesenchymal HGSOCs (Figure S11). However, we could only infer the association of these three genes, but not their causal relationship.

EMT is currently known as one of the cancer hallmarks, being involved in tumorigenesis, metastasis, and obtaining chemoresistance [11,13,39,40]. Unlike in breast cancer, the link between BRCA1 and EMT has not been investigated in HGSOC. The relationship between expression profiles of HRR and EMT genes might be explained by the following hypotheses: (1) the co-existence of deficient *BRCA1/2* or HRR genes and altered expression of EMT genes together lead cancer cells to extinction; or (2) altered expression of EMT genes may contribute to the tumor microenvironment being nonviable for cancer cells with defects in *BRCA1/2* or HRR genes. To confirm these hypotheses, additional experiments using ovarian cancer cell lines are warranted.

In the current study, we invented the EMT index, composed of 38 genes—five for core EMT-TFs and 33 for EMT-related TFs—which can be utilized in identifying mesenchymal HGSOC. In addition, it may be used as a prognostic marker in HGSOC; both in our samples and TCGA HGSOC data, a high EMT index was associated with significantly worse OS. At the same time, it should be noted that the proportion of stromal cells within samples might be reflected in the EMT index. Indeed, a higher proportion of stromal cells in HGSOC is known to be associated with worse OS [41]. Furthermore, various molecules, such as E-cadherin, N-cadherin, EpCAM, and vimentin, are involved in the EMT process [11]. A complex network of TFs is known to regulate EMT, leading to the downregulation of epithelial genes and the upregulation of mesenchymal genes [11,42]. We also observed various molecules or genes related to the EMT index and regulators of EMT, including vimentin and TGFβ, which were differentially expressed between the two types of HGSOC.

In terms of anti-EMT therapy, TGFβ is one of the best-studied therapeutic targets in cancer. Phase I and II clinical trials of fresolimumab (a monoclonal anti-TGFβ antibody) have been conducted in renal cell carcinoma, melanoma, mesothelioma, and breast cancer [43,44,45]. In ovarian cancer, blockade of TGFβ signaling with antibodies reversed EMT in epithelial ovarian cancer ascites-derived cell spheroids [46] and increased platinum sensitivity in a xenograft mouse model [47]. More research is needed to elucidate the therapeutic strategy of anti-EMT therapies in HGSOC.

Based on our study results, if an individual is identified to have a high-EMT-index HGSOC, so poor prognosis is expected, clinicians might prescribe additional targeted agents (e.g., bevacizumab) more actively. Clinicians might also consider dose-dense chemotherapy or extended chemotherapy cycles. After primary treatment, a more intensive surveillance schedule might be administered for an individual. Incorporating the EMT index with the well-known clinicopathologic risk factors of HGSOC, researchers might develop models predicting treatment response and prognosis more accurately. In this manner, we believe that precision cancer medicine can be facilitated in ovarian cancer with a relatively poorer prognosis than any other cancer.

Our study has several limitations. First, the small sample size might be one of the most problematic issues. In survival analysis, we could not conduct multivariate analysis adjusting for clinicopathologic factors. Thus, our study results should be validated in a large, multi-institutional HGSOC cohort. Second, our study results were only derived from bulky specimens composed of various malignant and non-malignant cells. Therefore, specific gene signatures of the mesenchymal HGSOC samples might be a mixed result originating from malignant epithelial or mesenchymal cells and non-malignant cells, such as CAFs, endothelial cells, and immune cells [29]. To elucidate the exact cellular compositions and heterogeneity in tumor cells, as well as the cell-to-cell interactions within the tumor microenvironment, further singe-cell-level studies should be conducted. Such studies might supplement and enhance our study results. Nevertheless, we believe that the methodology of our study, especially the step-by-step integrative analysis methods, can be also used in other malignancy types.

## 5. Conclusions

In conclusion, we investigated the molecular characteristics of HGSOC by utilizing exome and transcriptome sequencing data. Two distinctive types of HGSOC (HRR-activated and mesenchymal) were identified, which could be helpful for personalized HGSOC treatment. Furthermore, our novel EMT index seems to be a potential prognostic biomarker for HGSOC. Patients with high-EMT-index tumors showed significantly worse OS than those with low-EMT-index tumors. As such, molecules or genes related to the EMT index can be therapeutic targets for the treatment of HGSOC.

## Figures and Tables

**Table 1:** Patients’ clinicopathologic characteristics.

Characteristics	All (*n* = 20, %)	*BRCA* Mutation (*n* = 10, %)	*BRCA* Wild-Type (*n* = 10, %)	*p*
Age, years				
Mean ± SD	52.8 ± 8.4	54.2 ± 9.4	51.4 ± 7.4	0.705
Family Hx of breast cancer	1 (5.0)	1 (10.0)	0	>0.999
Family Hx of ovarian cancer	1 (5.0)	1 (10.0)	0	>0.999
FIGO stage				0.779
IIIA	2 (10.0)	1 (10.0)	1 (10.0)	
IIIB	1 (5.0)	1 (10.0)	0	
IIIC	11 (55.0)	5 (50.0)	6 (60.0)	
IV	6 (30.0)	3 (30.0)	3 (30.0)	
CA-125, IU/mL				
Median (range)	798.5 (5.1–3545.0)	798.0 (5.1–3545.0)	798.5 (47.0–2433.0)	0.940
Lymph node metastasis	12 (60.0)	6 (60.0)	6 (60.0)	>0.999
Residual tumor after surgery				0.139
No gross	14 (70.0)	9 (90.0)	5 (50.0)	
<1 cm	5 (25.0)	1 (10.0)	4 (40.0)	
≥1 and <2 cm	1 (5.0)	0	1 (10.0)	
Chemotherapy at primary treatment				0.628
6 cycles of paclitaxel–carboplatin	14 (70.0)	6 (60.0)	8 (80.0)	
9 cycles of paclitaxel–carboplatin	6 (30.0)	4 (40.0)	2 (20.0)	
Recurrence	16 (80.0)	9 (90.0)	7 (70.0)	0.582
Treatment-free interval, months				
Median (range)	20.4 (3.0–73.0)	20.9 (13.5–73.0)	19.6 (3.0–67.9)	0.496
Germline *BRCA1* mutational status				0.033
Wild-type	15 (75.0)	5 (50.0)	10 (100.0)	
Mutation	5 (25.0)	5 (50.0)	0	
Germline *BRCA2* mutational status				0.033
Wild-type	15 (75.0)	5 (50.0)	10 (100.0)	
Mutation	5 (25.0)	5 (50.0)	0	

Abbreviations: CA-125, cancer antigen 125; FIGO, International Federation of Gynecology and Obstetrics; Hx, history; SD, standard deviation.

## Data Availability

The sequence data were deposited in the Sequence Read Archive (SRA). The SRA accession number as well as codes and algorithms implemented in this study are available in Github at https://github.com/ryansohny/HGSOC. The data presented in this study are also available on request from the corresponding authors.

## Supplementary Materials

The following are available online at https://www.mdpi.com/article/10.3390/genes12071103/s1, Supplementary Methods, Table S1: A detailed summary of sequencing coverage and quality metrics of WES, Table S2: A detailed summary of sequencing coverage and quality metrics of RNA-seq, Table S3: List of pathogenic germline variants in *BRCA1* and *BRCA2* from blood samples of g*BRCA1*mut and g*BRCA2*mut groups, Table S4: List of genes positively correlated and negatively correlated with PC1, Table S5: TFEA results from genes negatively correlated with PC1 (Pearson r < −0.9), Table S6: List of 30 homologous recombination repair genes used in this study, Table S7: TFEA results from 18 out of 30 HRR genes upregulated in cluster A compared to those in cluster B, Table S8: TFEA results from genes positively correlated with PC1 (Pearson r > 0.9), Table S9: List of 38 genes used to calculate the EMT index, Table S10: Tumor purity and ploidy estimate for each sample, Figure S1: Germline *BRCA1/2* mutations across g*BRCA1/2*mut samples validated by whole-exome sequencing. IGV views (a copy number scatterplot for g*BRCA1/2*mut_5) of germline mutations across 10 g*BRCA1/2*mut samples show next-generation-sequencing-validated hemizygous mutations in the *BRCA1* or *BRCA2* gene, Figure S2: Boxplots showing TMBs across different groups of patients. There were no statistical differences in TMBs (one-way ANOVA, *p* = 0.313) among g*BRCA1*mut, g*BRCA2*mut, and g*BRCA1/2*wt samples. Each dot represents each TMB value of an HGSOC sample, while the average TMB values for each group are connected with a line. Boxplots show the 95% confidence interval for each group, Figure S3: Somatic copy number alteration profiles of 20 HGSOC samples. Oncoplot showing highly amplified and deleted genes. Each column represents an individual patient, Figure S4: Aberration of HRR genes across g*BRCA1/2*wt samples. The distribution of HRR gene alterations across 10 g*BRCA1/2*wt tumor samples. Each row corresponds to each tumor sample, and each row corresponds to an altered HRR gene, Figure S5: Boxplot showing the expression of TFs related to EMT across cluster A and cluster B. Boxplot shows the expression of EMT-related TF genes derived from TF enrichment analysis of genes displaying positive correlation (Pearson r > 0.9) with the PC1 value of the principal component analysis. Mann–Whitney U test *p* value <0.005 ** and *p* value < 0.05 *, Figure S6: Violin plot view of distribution of three HRD measures: loss of heterozygosity (LOH); number of telomeric allelic imbalances (NtAI); and large-scale transition (LST), Figure S7: Co-expression modules for cluster A and cluster B. The dot plot in the top right shows the module activity of each module in terms of normalized enrichment score (NES). The size of the circle represents the intensity of NES. The expression profiles of individual genes across our samples from cluster A and cluster B were visualized for Co-expression Module 1 (upper right), Co-expression Module 2 (lower left), and Co-expression Module 3 (lower right), Figure S8: Correlation between PC1 and tumor purity. Significant negative correlation between PC1 from RNA-seq and tumor purity derived from whole-exome sequencing (Pearson r = −0.843 and *p* < 0.001). PC, principal component, Figure S9: Expression of two CAF marker genes (*DCN* and *PDPN*). Violin plot showing the expression of two CAF marker genes for cluster A and cluster B. Mann–Whitney U test *p* value for each observation is represented above, Figure S10: Comparisons of survival outcomes between homologous recombination repair-activated and mesenchymal types. (A) Progression-free survival. (B) Overall survival, Figure S11: Expression of genes shaping the β-catenin/TCF4 complex and its downstream target *ZEB1* gene. Violin plot showing the expression of three genes, two of which together encoding the β-catenin/TCF4 complex (*CTNNB1* and *TCF4*) and one encoding zinc finger E-box-binding homeobox 1 (*ZEB1*).
