# Supplementary material for: Classification of High-Grade Serous Ovarian Carcinoma by Epithelial-to-Mesenchymal Transition Signature and Homologous Recombination Repair Genes
Source: Genes (Basel). 2021 Jul 20;12(7):1103. doi: 10.3390/genes12071103 (PMC8303300; doi:10.3390/genes12071103)

Fig. S1

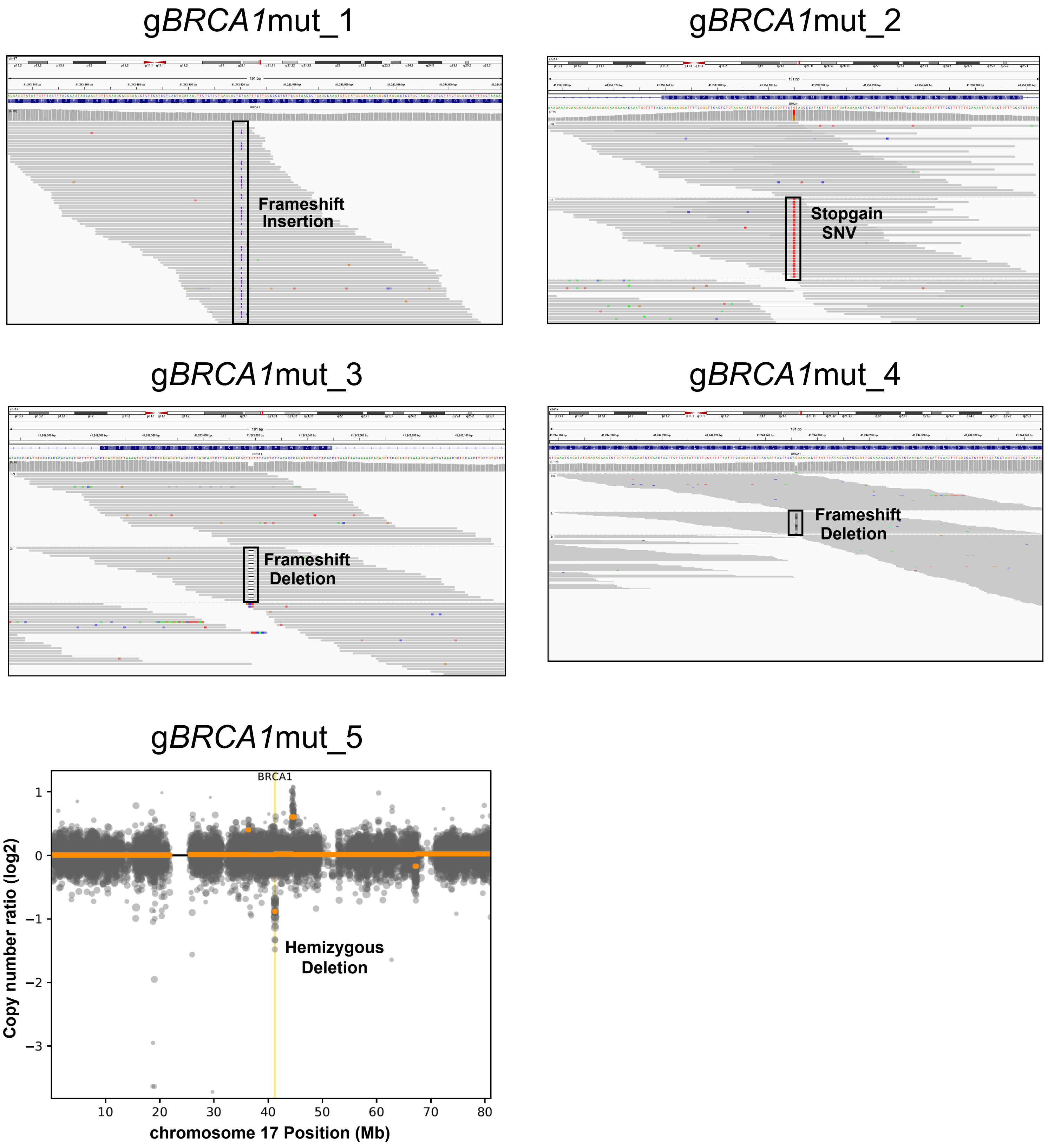
gBRCA1mut_1
Frameshift Insertion
gBRCA1mut_2
Stopgain SNV
gBRCA1mut_3
Frameshift Deletion
gBRCA1mut_4
Frameshift Deletion
gBRCA1mut_5
BRCA1
Hemizygous Deletion
Copy number ratio (log2)
1
0
−1
−2
−3
10
20
30
40
50
60
70
80
chromosome 17 Position (Mb)

Fig. S1

g*BRCA2*mut_1

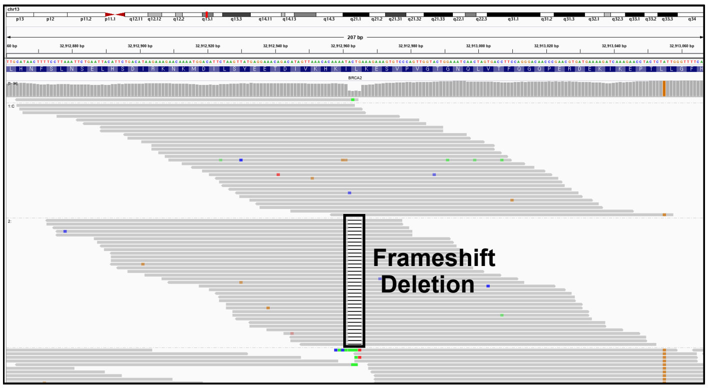

g*BRCA2*mut_2

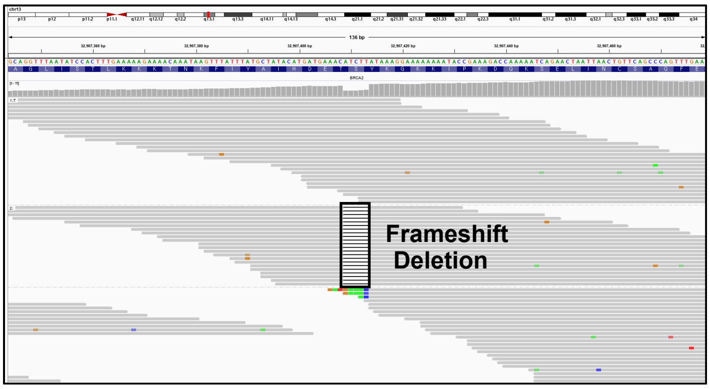

g*BRCA2*mut_3

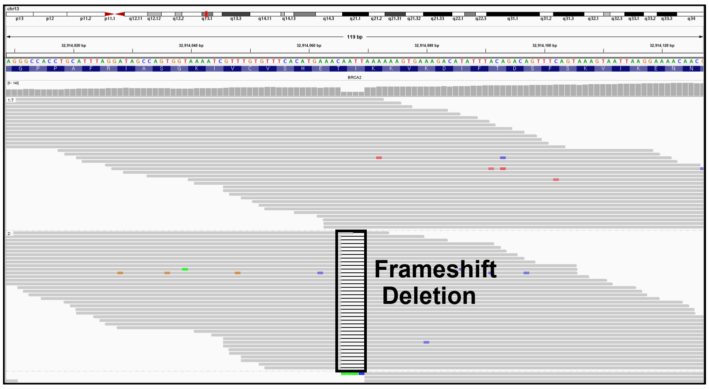

g*BRCA2*mut_4

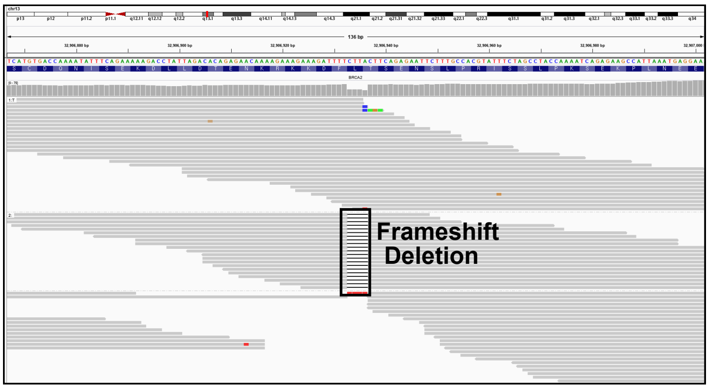

g*BRCA2*mut_5

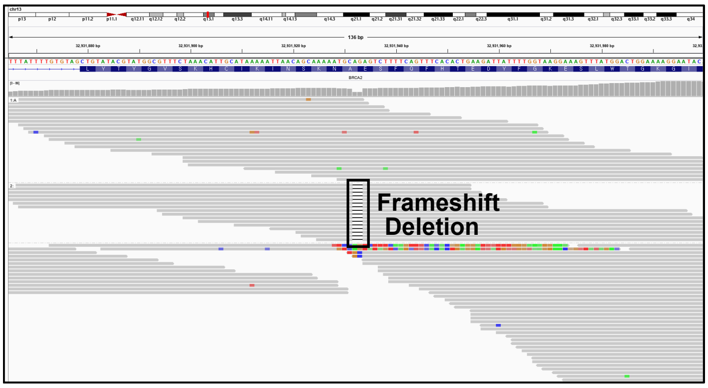

Supplement: Supplementary file 1 [file genes-12-01103-s001.zip › Figure S1.pdf]
