# Supplementary material for: Classification of High-Grade Serous Ovarian Carcinoma by Epithelial-to-Mesenchymal Transition Signature and Homologous Recombination Repair Genes
Source: Genes (Basel). 2021 Jul 20;12(7):1103. doi: 10.3390/genes12071103 (PMC8303300; doi:10.3390/genes12071103)
Supplement: Supplementary file 1 [file genes-12-01103-s001.zip › Supplementary Methods.pdf]

Supplementary Methods

Whole-exome library preparation and sequencing

First, we obtained DNA from fresh-frozen, chemotherapy-naïve, primary ovarian cancer tissue samples and matched normal blood samples using Puregene Core kit and QIAamp DNA Blood Mini kit, respectively. Then, we captured human exon regions using SureSelect Human All Exon V6 kit following standard protocols. Subsequently, we performed 101X2 paired-end WES using HiSeq2500 instrument (Illumina, San Diego, CA, USA), according to the manufacturer's instruction.

Whole exome sequencing data pre-processing and variant detection

Raw FASTQ files were aligned onto GRCh37 using Burrow-wheeler Aligner (BWA) mem algorithms [1], and the resulting bam files were subjected to duplicate removal using the Genome Analysis Tool Kit (GATK) version 4.1.4.1 [2]. After base quality score recalibration and applying it to each bam file using GATK, we proceeded to the discovery of somatic single nucleotide variants (SNVs) and small insertions and deletions (indels) for each tumor sample using Strelka2 [3] and a paired-normal sample as a control. For germline variant discovery of the WES from normal samples, we applied GATK's HaplotypeCaller. All of the above variants were annotated by Oncotator [4] and ANNOVAR [5]. To accurately pinpoint the actually harmful ones, we only retained exonic variants (i.e. Missense, Nonsense, Frameshift insertion, Frameshift deletion, In-frame insertion, In-frame deletion and Splice site mutation) with at least 10x coverage of alternate allele, predicted to be deleterious by SIFT [6] and having minor allele frequency below 0.1% in 1000 Genomes Project phase 3 data [7], EXome Aggregation Consortium data [8], and Northeast Asian Reference Database [9]. Then, we only kept genes that are overlapped with cancer consensus genes from the Catalogue of Somatic Mutations in Cancer database [10]. Tumor mutational burden (TMB) was estimated by the total somatic mutations for each sample divided by the length of the captured exon regions (61 Mb).

Copy number alteration detection

In order to discover somatic copy number alterations (SCNAs), we used CNVkit with default parameters [11]. Specifically, bin-level log2 ratio (.cnr) and segmented log2 ratio (.cns) files, generated from bam files by a separate reference for each matched tumor-normal pair, were processed into the residual bin-level log2 ratio estimates (segmetrics command). Then, GISTIC2 [12] was implemented to identify frequently altered chromosomal regions with a confidence level of 0.90 and a Q-value threshold of 0.05. The purity of the tumor samples was calculated via Sequenza algorithm [13]. We also used seqz files generated by Sequenza as an input to scarHRD [14] for HRD score estimation. To discover germline SCNAs by using the CNVkit, we constructed a pooled reference from 20 normal blood samples and followed the same approach as that used for detecting somatic SCNAs using the CNVkit.

RNA sequencing and data analysis

We extracted RNA from primary ovarian cancer tissue samples and prepared sequencing library using TruSeq RNA Access Library Prep Kit under standard protocol. Then we conducted RNA-seq on 20 HGSOC tumor samples by 101X2 paired-end mode using Illumina HiSeq2500 (Illumina, San Diego, California), in accordance with the manufacturer's instruction. For RNA-seq data analysis, each transcript expression was first quantified by pseudoalignment algorithm implicated in kallisto [15] version 0.46.1 using RefSeq annotation release 105 for GRCh37. Quantified transcript-level transcripts per million (TPM) values were collapsed to give gene-level expression, and only the protein-coding genes were processed for the rest of the analysis. TPM values were implemented for comparison among different groups and for inputs to cell type enrichment analysis. With regard to discovering differentially expressed genes (DEG) among sample groups ((i)

g*BRCA1*mut, g*BRCA2*mut and g*BRCA1/2*wt and (ii) HRR-activated and mesenchymal), we used DESeq2 [16] version 1.24.0. Raw counts of the RNA-seq were transformed using variance stabilizing transformations, which were later used as inputs to principal component analysis (PCA), unsupervised hierarchical clustering (HC) and identification of gene co-expression modules and interaction networks. With respect to PCA, the top 5,000 variable genes among 19,023 genes were used as inputs. Samples were then grouped into two clusters according to K-means clustering with k=2. For HC, we used Euclidean distance measure and uncentered correlation measure for EMT transcription factors (EMT-TFs) and homologous recombination repair (HRR) genes, respectively. Each gene expression was centered to the average intensity of samples, along with pair-wise complete-linkage for clustering of both samples and genes using Cluster 3.0 [17]. We visualized the resulting distance measures and dendrograms through Java Treeview [18].

References

[1] Li H, Durbin R. Fast and accurate short read alignment with Burrows-Wheeler transform. Bioinformatics. 2009;25:1754-60.

[2] DePristo MA, Banks E, Poplin R, Garimella KV, Maguire JR, Hartl C, et al. A framework for variation discovery and genotyping using next-generation DNA sequencing data. Nat Genet. 2011;43:491-8.

[3] Kim S, Scheffler K, Halpern AL, Bekritsky MA, Noh E, Kallberg M, et al. Strelka2: fast and accurate calling of germline and somatic variants. Nat Methods. 2018;15:591-4.

[4] Ramos AH, Lichtenstein L, Gupta M, Lawrence MS, Pugh TJ, Saksena G, et al. Oncotator: cancer variant annotation tool. Hum Mutat. 2015;36:E2423-9.

[5] Wang K, Li M, Hakonarson H. ANNOVAR: functional annotation of genetic variants from high-throughput sequencing data. Nucleic Acids Res. 2010;38:e164.

[6] Ng PC, Henikoff S. SIFT: Predicting amino acid changes that affect protein function. Nucleic Acids Res. 2003;31:3812-4.

[7] Genomes Project C, Auton A, Brooks LD, Durbin RM, Garrison EP, Kang HM, et al. A global reference for human genetic variation. Nature. 2015;526:68-74.

[8] Lek M, Karczewski KJ, Minikel EV, Samocha KE, Banks E, Fennell T, et al. Analysis of protein-coding genetic variation in 60,706 humans. Nature. 2016;536:285-91.

[9] Yoo SK, Kim CU, Kim HL, Kim S, Shin JY, Kim N, et al. NARD: whole-genome reference panel of 1779 Northeast Asians improves imputation accuracy of rare and low-frequency variants. Genome Med. 2019;11:64.

[10] Tate JG, Bamford S, Jubb HC, Sondka Z, Beare DM, Bindal N, et al. COSMIC: the Catalogue Of Somatic Mutations In Cancer. Nucleic Acids Res. 2019;47:D941-D7.

[11] Talevich E, Shain AH, Botton T, Bastian BC. CNVkit: Genome-Wide Copy Number Detection and Visualization from Targeted DNA Sequencing. PLoS Comput Biol. 2016;12:e1004873.

[12] Mermel CH, Schumacher SE, Hill B, Meyerson ML, Beroukhim R, Getz G. GISTIC2.0 facilitates sensitive and confident localization of the targets of focal somatic copy-number alteration in human cancers. Genome Biol. 2011;12:R41.

[13] Favero F, Joshi T, Marquard AM, Birkbak NJ, Krzystanek M, Li Q, et al. Sequenza: allele-specific copy number and mutation profiles from tumor sequencing data. Ann Oncol. 2015;26:64-70.

[14] Sztupinszki Z, Diossy M, Krzystanek M, Reiniger L, Csabai I, Favero F, et al. Migrating the SNP array-based homologous recombination deficiency measures to next generation sequencing data of breast cancer. NPJ Breast Cancer. 2018;4:16.

[15] Bray NL, Pimentel H, Melsted P, Pachter L. Near-optimal probabilistic RNA-seq quantification. Nat Biotechnol. 2016;34:525-7.

[16] Love MI, Huber W, Anders S. Moderated estimation of fold change and dispersion for RNA-seq data with DESeq2. Genome Biol. 2014;15:550.

[17] de Hoon MJ, Imoto S, Nolan J, Miyano S. Open source clustering software. Bioinformatics. 2004;20:1453-4.

[18] Saldanha AJ. Java Treeview--extensible visualization of microarray data. Bioinformatics. 2004;20:3246-8.
