# Supplementary material for: Classification of High-Grade Serous Ovarian Carcinoma by Epithelial-to-Mesenchymal Transition Signature and Homologous Recombination Repair Genes
Source: Genes (Basel). 2021 Jul 20;12(7):1103. doi: 10.3390/genes12071103 (PMC8303300; doi:10.3390/genes12071103)
Supplement: Supplementary file 1 [file genes-12-01103-s001.zip › Table S1.pdf]

Table S1. A detailed summary of sequencing coverage and quality metrics of WES.

Sample ID	Category	Total number of sequenced reads	Total number of mapped non-duplicate reads	Properly Paired reads	Mate mapped to a different chromosome	Mate mapped to a different chromosome with mapping quality ≥5	Total number of covered bases	Coverage per base, median (IQR)	Percentage of targeted bases with coverage ≥10	Percentage of targeted bases with coverage ≥20	Percentage of targeted bases with coverage ≥30
N_BRCA1_1	Normal WES	132,561,499	95,720,825	94,877,897	232,952	218,856	98,392,567	71 (34–121)	94.4	86.1	77.6
N_BRCA1_2	Normal WES	116,232,714	81,140,930	80,282,572	400,754	378,440	98,334,347	59 (28–102)	92.3	81.9	72.3
N_BRCA1_3	Normal WES	112,369,126	73,085,486	72,424,000	361,868	340,220	98,592,361	53 (30–85)	95.6	86.2	74.4
N_BRCA1_4	Normal WES	112,978,210	80,529,713	79,891,545	297,929	280,451	98,419,245	59 (30–99)	94	84.5	74.6
N_BRCA1_5	Normal WES	117,348,438	85,986,674	85,194,614	330,489	311,324	98,396,254	62 (29–107)	93.1	83.3	73.9
N_BRCA2_1	Normal WES	114,976,738	79,916,077	79,220,687	408,754	385,575	98,485,396	59 (31–97)	94.8	85.9	75.6
N_BRCA2_2	Normal WES	105,035,318	77,091,194	76,255,776	320,857	302,978	98,290,372	56 (26–98)	91.8	80.8	70.8
N_BRCA2_3	Normal WES	99,579,372	72,865,494	72,186,388	322,659	304,585	98,392,451	54 (27–91)	92.9	82.1	71.4
N_BRCA2_4	Normal WES	103,268,159	75,528,325	74,662,055	436,185	412,290	98,309,828	55 (26–97)	91.8	80.7	70.7
N_BRCA2_5	Normal WES	102,690,757	72,426,343	71,904,429	179,664	168,245	98,313,616	53 (26–92)	92.2	81	70.5
N_WT_1	Normal WES	109,730,922	78,977,243	78,217,563	384,152	362,312	98,390,936	57 (28–99)	92.9	82.6	72.6
N_WT_2	Normal WES	102,006,034	72,768,774	72,166,118	345,855	325,362	98,478,000	53 (28–89)	94.5	83.8	72.5
N_WT_3	Normal WES	111,474,711	79,702,748	79,062,015	261,611	246,235	98,403,892	60 (30–100)	94.2	84.8	74.9
N_WT_4	Normal WES	102,282,842	70,904,467	70,160,093	278,221	262,197	98,316,572	52 (25–90)	91.5	79.8	69.2
N_WT_5	Normal WES	101,865,324	74,586,718	73,945,627	237,280	223,202	98,371,588	55 (27–95)	92.4	81.4	71
N_WT_6	Normal WES	104,086,149	75,146,531	74,523,164	204,264	192,154	98,336,341	56 (27–96)	92.5	81.7	71.6
N_WT_7	Normal WES	104,994,622	77,145,337	76,484,121	201,499	189,437	98,290,628	56 (26–96)	91.8	80.7	70.7
N_WT_8	Normal WES	95,900,638	68,999,557	68,157,536	383,149	362,449	98,224,123	49 (22–86)	89.7	77.2	66.6
N_WT_9	Normal WES	100,755,435	71,809,823	71,156,885	241,262	227,374	98,291,743	52 (25–89)	91.8	80.2	69.6
N_WT_10	Normal WES	100,635,873	71,699,846	71,004,355	235,204	222,146	98,266,601	52 (24–90)	90.9	79.1	68.6
T_BRCA1_1	Tumor WES	153,482,549	111,415,410	110,001,095	508,800	480,413	98,366,775	77 (36–138)	94.2	86.5	78.7
T_BRCA1_2	Tumor WES	145,899,568	104,528,168	103,491,717	345,480	324,526	98,405,326	70 (33–126)	93.8	85.5	77.1
T_BRCA1_3	Tumor WES	135,408,443	99,561,085	98,505,640	350,595	330,401	98,395,824	69 (32–122)	93.7	85	76.4
T_BRCA1_4	Tumor WES	136,717,978	101,467,903	100,381,978	307,259	288,788	98,399,152	68 (32–123)	93.5	84.9	76.3
T_BRCA1_5	Tumor WES	146,203,797	104,664,776	103,646,246	334,561	314,758	98,405,083	71 (33–128)	93.7	85.5	77.1
T_BRCA2_1	Tumor WES	150,906,453	102,105,761	100,896,933	491,939	464,858	98,372,723	70 (32–126)	93.4	84.8	76.4
T_BRCA2_2	Tumor WES	149,918,478	109,969,198	108,921,373	353,418	333,402	98,387,024	76 (36–135)	94.4	86.7	78.7
T_BRCA2_3	Tumor WES	138,000,700	103,621,959	102,490,195	408,502	385,843	98,391,798	74 (34–130)	94.1	86	77.9
T_BRCA2_4	Tumor WES	146,155,430	106,231,786	105,202,298	357,994	337,327	98,395,491	75 (35–131)	94.3	86.6	78.6
T_BRCA2_5	Tumor WES	144,780,721	103,143,893	102,020,162	413,441	389,099	98,382,112	69 (33–125)	93.8	85.5	76.9
T_WT_1	Tumor WES	140,166,492	102,205,335	101,141,107	333,806	314,492	98,422,545	72 (34–126)	94.2	86.2	77.9

T_WT_2	Tumor WES	143,237,732	104,389,002	103,447,240	309,711	291,196	98,322,446	70 (32–127)	93.2	84.6	76.3
T_WT_3	Tumor WES	142,754,271	100,362,774	99,266,080	383,073	360,988	98,333,642	70 (32–125)	93.6	85	76.5
T_WT_4	Tumor WES	149,009,652	102,660,646	101,782,136	251,625	235,866	98,348,541	72 (33–127)	93.8	85.1	76.9
T_WT_5	Tumor WES	146,657,432	106,445,024	105,276,908	427,012	401,926	98,387,584	72 (34–130)	93.8	85.6	77.5
T_WT_6	Tumor WES	147,477,186	107,688,197	106,637,986	349,053	329,270	98,388,516	78 (36–136)	94.6	87.1	79.2
T_WT_7	Tumor WES	150,076,192	108,436,494	107,324,481	330,318	310,294	98,426,389	78 (37–135)	94.9	87.6	79.8
T_WT_8	Tumor WES	139,283,195	101,507,796	100,354,292	414,630	390,862	98,409,057	71 (33–126)	93.9	85.6	77.3
T_WT_9	Tumor WES	144,984,211	101,593,268	100,653,016	325,161	306,343	98,358,925	70 (32–126)	93.6	85	76.5
T_WT_10	Tumor WES	152,112,193	110,081,078	109,088,724	242,472	227,047	98,388,241	72 (33–133)	93.5	85.1	76.9

Abbreviation: IQR, interquartile range; WES, whole exome sequencing.
Whole Exome Sequencing reads (captured by SureSelect Human All Exon V6 kit) aligned onto GRCh37 reference genome using BWA aligner.
