# Supplementary material for: Classification of High-Grade Serous Ovarian Carcinoma by Epithelial-to-Mesenchymal Transition Signature and Homologous Recombination Repair Genes
Source: Genes (Basel). 2021 Jul 20;12(7):1103. doi: 10.3390/genes12071103 (PMC8303300; doi:10.3390/genes12071103)
Supplement: Supplementary file 1 [file genes-12-01103-s001.zip › Table S10.pdf]

Table S10. Tumor purity and ploidy estimate for each sample.

Sample	Tumor Purity	Ploidy
g*BRCA1*mut_1	0.44	3.697
g*BRCA1*mut_2	0.77	2.679
g*BRCA1*mut_3	0.50	3.728
g*BRCA1*mut_4	0.73	3.307
g*BRCA1*mut_5	0.77	5.886
g*BRCA2*mut_1	0.58	2.350
g*BRCA2*mut_2	0.48	3.511
g*BRCA2*mut_3	0.10	2.010
g*BRCA2*mut_4	0.51	3.122
g*BRCA2*mut_5	0.75	1.913
g*BRCA1/2w*t_1	0.43	1.949
g*BRCA1/2w*t_2	0.88	3.350
g*BRCA1/2w*t_3	0.95	1.864
g*BRCA1/2w*t_4	0.10	2.110
g*BRCA1/2w*t_5	0.80	3.518
g*BRCA1/2w*t_6	0.31	2.001
g*BRCA1/2w*t_7	0.10	2.060
g*BRCA1/2w*t_8	0.43	3.420
g*BRCA1/2w*t_9	0.72	3.903
g*BRCA1/2w*t 10	0.86	1.794
