# Supplementary material for: Classification of High-Grade Serous Ovarian Carcinoma by Epithelial-to-Mesenchymal Transition Signature and Homologous Recombination Repair Genes
Source: Genes (Basel). 2021 Jul 20;12(7):1103. doi: 10.3390/genes12071103 (PMC8303300; doi:10.3390/genes12071103)
Supplement: Supplementary file 1 [file genes-12-01103-s001.zip › Table S2.pdf]

Table S2. A detailed summary of sequencing coverage and quality metrics of RNA-seq.

Sample ID	Category	Total number of sequenced reads	Total number of pseudoaligned reads*	Total number of uniquely pseudoaligned reads	Percentage of pseudoaligned reads	Percentage of uniquely pseudoaligned reads	rRNA rate
T_BRCA1_1	Tumor RNA	55,078,344	51,890,442	19,515,066	94.2	35.4	8.84
T_BRCA1_2	Tumor RNA	57,434,868	55,494,782	20,026,139	96.6	34.9	2.24
T_BRCA1_3	Tumor RNA	54,152,977	51,418,251	18,425,455	94.9	34	4.27
T_BRCA1_4	Tumor RNA	52,514,023	49,118,309	17,913,500	93.5	34.1	8.39
T_BRCA1_5	Tumor RNA	58,060,461	55,652,375	20,104,418	95.9	34.6	4.66
T_BRCA2_1	Tumor RNA	51,043,293	48,135,468	15,890,054	94.3	31.1	14.80
T_BRCA2_2	Tumor RNA	59,476,054	55,642,619	19,924,445	93.6	33.5	15.14
T_BRCA2_3	Tumor RNA	57,676,005	54,661,802	18,441,833	94.8	32	10.30
T_BRCA2_4	Tumor RNA	57,221,098	54,660,071	20,239,754	95.5	35.4	8.06
T_BRCA2_5	Tumor RNA	59,061,460	56,680,666	20,376,798	96	34.5	5.59
T_WT_1	Tumor RNA	58,764,960	56,240,783	21,923,911	95.7	37.3	6.77
T_WT_2	Tumor RNA	58,938,404	57,253,142	21,881,071	97.1	37.1	2.53
T_WT_3	Tumor RNA	56,121,852	53,846,348	18,892,959	95.9	33.7	3.89
T_WT_4	Tumor RNA	58,300,346	53,742,473	24,310,535	92.2	41.7	11.62
T_WT_5	Tumor RNA	60,704,131	57,223,716	19,859,280	94.3	32.7	10.83
T_WT_6	Tumor RNA	55,430,685	52,525,610	18,441,119	94.8	33.3	9.45
T_WT_7	Tumor RNA	61,660,304	58,500,190	21,383,314	94.9	34.7	9.52
T_WT_8	Tumor RNA	58,243,025	55,325,870	21,029,168	95	36.1	9.20
T_WT_9	Tumor RNA	60,054,479	57,534,380	22,098,744	95.8	36.8	3.24
T_WT_10	Tumor RNA	55,988,981	53,477,201	21,509,238	95.5	38.4	1.83

Abbreviation: RNA-seq, RNA sequencing.
*Pseudoaligned using kallisto onto RefSeq annotation release 105 for GRCh37.
