# Supplementary material for: Classification of High-Grade Serous Ovarian Carcinoma by Epithelial-to-Mesenchymal Transition Signature and Homologous Recombination Repair Genes
Source: Genes (Basel). 2021 Jul 20;12(7):1103. doi: 10.3390/genes12071103 (PMC8303300; doi:10.3390/genes12071103)
Supplement: Supplementary file 1 [file genes-12-01103-s001.zip › Table S4.pdf]

Table S4. List of genes positively correlated and negatively correlated with PC1.

Genes positively correlated with PC1 (Pearson r>0.9)	Genes negatively correlated with PC1 (Pearson r<-0.9)
FABP4 (0.973)	BAIAP2L1 (-0.969)
CCDC36 (0.971)	KDF1 (-0.967)
GNG11 (0.971)	TPD52 (-0.951)
CPED1 (0.969)	GRHL2 (-0.949)
ECM2 (0.965)	C19orf33 (-0.945)
LRRC17 (0.965)	HOOK1 (-0.942)
CD302 (0.963)	FOLR1 (-0.942)
ZNF781 (0.963)	MARVELD2 (-0.942)
FHL5 (0.962)	ESRP1 (-0.941)
PLA2G5 (0.961)	EPPK1 (-0.939)
LIX1L (0.960)	PTGS1 (-0.938)
ITM2A (0.960)	MAL2 (-0.938)
JAM2 (0.958)	ARHGAP8 (-0.937)
GRK5 (0.957)	PARD6B (-0.936)
DGKB (0.955)	SMPDL3B (-0.929)
COX7A1 (0.953)	EPCAM (-0.928)
ENPP6 (0.953)	SPINT1 (-0.928)
RERGL (0.952)	TMPRSS3 (-0.928)
STXBP5L (0.952)	MYEF2 (-0.928)
TMEM150C (0.951)	CHD7 (-0.927)
SOCS2 (0.951)	MUC1 (-0.926)
ITPR1 (0.950)	AP1M2 (-0.926)
DIRAS3 (0.950)	IGSF9 (-0.924)
TTYH2 (0.949)	CLDN7 (-0.923)
LRRN4CL (0.948)	RCC1 (-0.922)
WFIKKN2 (0.948)	FAM83H (-0.920)
NUDT11 (0.947)	SCNN1A (-0.918)
CCL14 (0.947)	MYH14 (-0.918)
CNRIP1 (0.947)	CBLC (-0.917)
OGN (0.947)	MSLN (-0.916)
PRPH2 (0.946)	OVOL2 (-0.916)
FGF2 (0.946)	MUC16 (-0.916)
AMELX (0.946)	C6orf132 (-0.915)
RAB9B (0.945)	PAX8 (-0.915)
PRSS35 (0.945)	ZNF165 (-0.914)
CSDC2 (0.945)	EPS8L1 (-0.913)
XPNPEP2 (0.945)	ILDR1 (-0.913)
PLP1 (0.944)	KLHL14 (-0.913)
SYNE1 (0.944)	EHF (-0.912)
PDE1C (0.943)	WWC1 (-0.910)
NEGR1 (0.943)	CAMSAP3 (-0.909)
ANKRD34A (0.941)	SPINT2 (-0.908)
TRPC6 (0.941)	ESRP2 (-0.908)
ANGPTL1 (0.941)	DDR1 (-0.907)
CTNNA3 (0.940)	CELSR1 (-0.907)
GPRASP1 (0.940)	ABHD11 (-0.907)
LGI4 (0.939)	CELSR2 (-0.907)
HAND2 (0.939)	SERINC2 (-0.906)

NID1 (0.939)
NDNF (0.938)
NR4A3 (0.938)
PDZRN4 (0.938)
RNASE4 (0.937)
GPRASP2 (0.937)
LRRTM3 (0.937)
MMRN1 (0.937)
SNCA (0.937)
PROK1 (0.937)
TCEAL7 (0.936)
PPARG (0.936)
ADH1B (0.936)
C11orf96 (0.936)
BTG2 (0.933)
PRDM8 (0.933)
SLC7A14 (0.933)
LEFTY2 (0.932)
STAB2 (0.931)
DPT (0.931)
NAV3 (0.931)
PAPSS2 (0.931)
RARA (0.930)
TSPAN5 (0.929)
SLC18A2 (0.929)
ABCA8 (0.928)
TWIST2 (0.928)
NDN (0.927)
SPARCL1 (0.927)
ARHGAP6 (0.927)
PSD3 (0.926)
VIT (0.926)
GSTM5 (0.926)
SPRR2F (0.926)
PLD5 (0.926)
ADRB3 (0.926)
KLF2 (0.925)
MFAP4 (0.925)
CELF2 (0.925)
STAT5B (0.924)
ZNF331 (0.924)
PDE1B (0.923)
RHOBTB3 (0.923)
MALRD1 (0.923)
PTGFR (0.923)
NR2F1 (0.922)
GNA14 (0.922)
MYH11 (0.922)
FSHR (0.921)
PABPC5 (0.921)
GEM (0.920)

CLDN3 (-0.905)
KLK8 (-0.904)
RHPN1 (-0.904)
CRB3 (-0.904)
VAMP8 (-0.903)
GRB7 (-0.903)
RASAL1 (-0.903)
PKP3 (-0.902)
LSR (-0.902)
SLC29A2 (-0.902)
BCAM (-0.902)
S100A14 (-0.900)

CPZ (0.920)
MAB21L1 (0.919)
CCN5 (0.919)
KLF9 (0.919)
VAT1 (0.918)
NXPH3 (0.918)
SCUBE1 (0.918)
CLEC3B (0.917)
TENT5A (0.917)
HRC (0.916)
KLF4 (0.916)
KIAA1755 (0.916)
SYTL4 (0.916)
ITGA8 (0.916)
FOXO1 (0.916)
PEAR1 (0.915)
ANTXR2 (0.915)
CALCRL (0.915)
RAB3C (0.914)
HIC1 (0.914)
CPEB1 (0.913)
CIRBP (0.913)
ZCCHC18 (0.913)
ANKRD29 (0.913)
VIM (0.912)
PROS1 (0.911)
TPSG1 (0.911)
ACSM2B (0.910)
CRHBP (0.910)
RHOB (0.909)
LIN7A (0.909)
CCBE1 (0.909)
GLP2R (0.909)
CD300LG (0.909)
NR1H4 (0.908)
TRPC4 (0.908)
TFPI (0.908)
HAS1 (0.908)
SGCZ (0.908)
ZEB2 (0.907)
STAMBPL1 (0.907)
DLC1 (0.907)
CSRNP1 (0.907)
MCC (0.907)
PER1 (0.907)
STARD9 (0.907)
PCED1B (0.906)
FXYD2 (0.906)
C21orf62 (0.906)
ZBTB20 (0.906)
C3orf70 (0.905)

ANG (0.905)
C8orf49 (0.905)
PCDH18 (0.905)
ARHGAP10 (0.905)
FEZ1 (0.905)
LHFPL6 (0.905)
KLHL4 (0.904)
PODN (0.904)
BMPER (0.904)
MORF4L2 (0.904)
GLI2 (0.904)
ARHGEF6 (0.904)
ARMCX4 (0.904)
TNFSF12 (0.903)
CDON (0.903)
ANGPT4 (0.903)
IGFBP4 (0.903)
PLD1 (0.903)
PRDM6 (0.903)
PLPP3 (0.903)
FGF7 (0.902)
CLDN11 (0.902)
SLC1A7 (0.902)
GNA12 (0.902)
PLBD2 (0.901)
STARD13 (0.901)
NAP1L3 (0.900)
MRGPRF (0.900)
SOBP (0.900)
GFRA1 (0.900)

Abbreviation: PC, principal component.
