# Supplementary material for: Classification of High-Grade Serous Ovarian Carcinoma by Epithelial-to-Mesenchymal Transition Signature and Homologous Recombination Repair Genes
Source: Genes (Basel). 2021 Jul 20;12(7):1103. doi: 10.3390/genes12071103 (PMC8303300; doi:10.3390/genes12071103)
Supplement: Supplementary file 1 [file genes-12-01103-s001.zip › Table S5.pdf]

Table S5. TFEA results from genes negatively correlated with PC1 (Pearson r <-0.9).

Transcription Factor	Overlapping Genes	FDR *Q* value
GRHL2	28	4.02E-27
SPDEF	27	4.85E-26
FOXA1	26	7.58E-25
OVOL1	24	1.91E-22
ELF3	24	1.91E-22
IRF6	24	1.91E-22
EHF	23	3.50E-21
KLF5	22	5.07E-20
GATA3	22	5.07E-20
TFCP2L1	22	5.07E-20
