# Supplementary material for: Classification of High-Grade Serous Ovarian Carcinoma by Epithelial-to-Mesenchymal Transition Signature and Homologous Recombination Repair Genes
Source: Genes (Basel). 2021 Jul 20;12(7):1103. doi: 10.3390/genes12071103 (PMC8303300; doi:10.3390/genes12071103)
Supplement: Supplementary file 1 [file genes-12-01103-s001.zip › Table S6.pdf]

Table S6. List of 30 homologous recombination repair genes used in this study.

Homologous recombination repair genes
BRCA1
BRCA2
ATM
ATR
ATRX
BARD1
BLM
BRIP1
CHEK1
CHEK2
FANCA
FANCC
FANCD2
FANCE
FANCF
FANCG
FANCI
FANCL
FANCM
MRE11
NBN
PALB2
RAD50
RAD51
RAD51B
RAD51C
RAD51D
RAD52
RAD54L
RPA1
