# Supplementary material for: Classification of High-Grade Serous Ovarian Carcinoma by Epithelial-to-Mesenchymal Transition Signature and Homologous Recombination Repair Genes
Source: Genes (Basel). 2021 Jul 20;12(7):1103. doi: 10.3390/genes12071103 (PMC8303300; doi:10.3390/genes12071103)
Supplement: Supplementary file 1 [file genes-12-01103-s001.zip › Table S7.pdf]

Table S7. TFEA results from 18 out of 30 HRR genes upregulated in cluster A compared to those in cluster B.

Transcription Factor	Overlapping Genes	FDR *Q* value
E2F8	8	4.18E-06
E2F2	6	8.68E-04
ZNF227	4	4.10E-02
ZNF107	4	4.10E-02
PRDM9	4	4.10E-02
ZNF45	4	4.10E-02
E2F3	4	4.10E-02
CENPA	4	4.10E-02
ZNF689	4	4.10E-02
TGIF2	4	4.10E-02
