# Supplementary material for: Classification of High-Grade Serous Ovarian Carcinoma by Epithelial-to-Mesenchymal Transition Signature and Homologous Recombination Repair Genes
Source: Genes (Basel). 2021 Jul 20;12(7):1103. doi: 10.3390/genes12071103 (PMC8303300; doi:10.3390/genes12071103)
Supplement: Supplementary file 1 [file genes-12-01103-s001.zip › Table S8.pdf]

Table S8. TFEA results from genes positively correlated with PC1 (Pearson r > 0.9).

Transcription Factor	Overlapping Genes	FDR *Q* value
TCF21	20	1.86E-08
TWIST2	17	3.11E-06
MEOX2	16	1.09E-05
OSR2	16	1.09E-05
OSR1	15	3.41E-05
PRRX1	15	3.41E-05
PRRX2	15	3.41E-05
BCL6B	14	1.35E-04
ATOH8	14	1.35E-04
TWIST1	14	1.35E-04
