# Supplementary material for: Classification of High-Grade Serous Ovarian Carcinoma by Epithelial-to-Mesenchymal Transition Signature and Homologous Recombination Repair Genes
Source: Genes (Basel). 2021 Jul 20;12(7):1103. doi: 10.3390/genes12071103 (PMC8303300; doi:10.3390/genes12071103)
Supplement: Supplementary file 1 [file genes-12-01103-s001.zip › Table S9.pdf]

Table S9. List of 38 genes used to calculate the EMT index.

Core EMT-TFs	Other EMT-related TFs
SNAI1	*KLF4*
SNAI2	*GSC*
ZEB1	*TCF7L2*
ZEB2	*ALX1*
TWIST1	*GATA6*
	RUNX2
	TCF3
	SOX4
	FOXC2
	NFKB1
	KLF2
	KLF6
	TBX3
	TCF4
	PRRX1
	HOXB7
	JUN
	FOS
	TAZ
	TGIF1
	ATF1
	ERG
	ETS1
	ID1
	TEAD1
	YAP1
	NFYA
	KLF8
	SOX9
	SIX1
	TBXT
	GATA4
	TWIST2

Abbreviations: EMT, epithelial-to-mesenchymal transition; TF, transcription factor.
