# Supplementary figures and images for: Classification of High-Grade Serous Ovarian Carcinoma by Epithelial-to-Mesenchymal Transition Signature and Homologous Recombination Repair Genes
Source: Genes (Basel). 2021 Jul 20;12(7):1103. doi: 10.3390/genes12071103 (PMC8303300; doi:10.3390/genes12071103)

Fig. S10

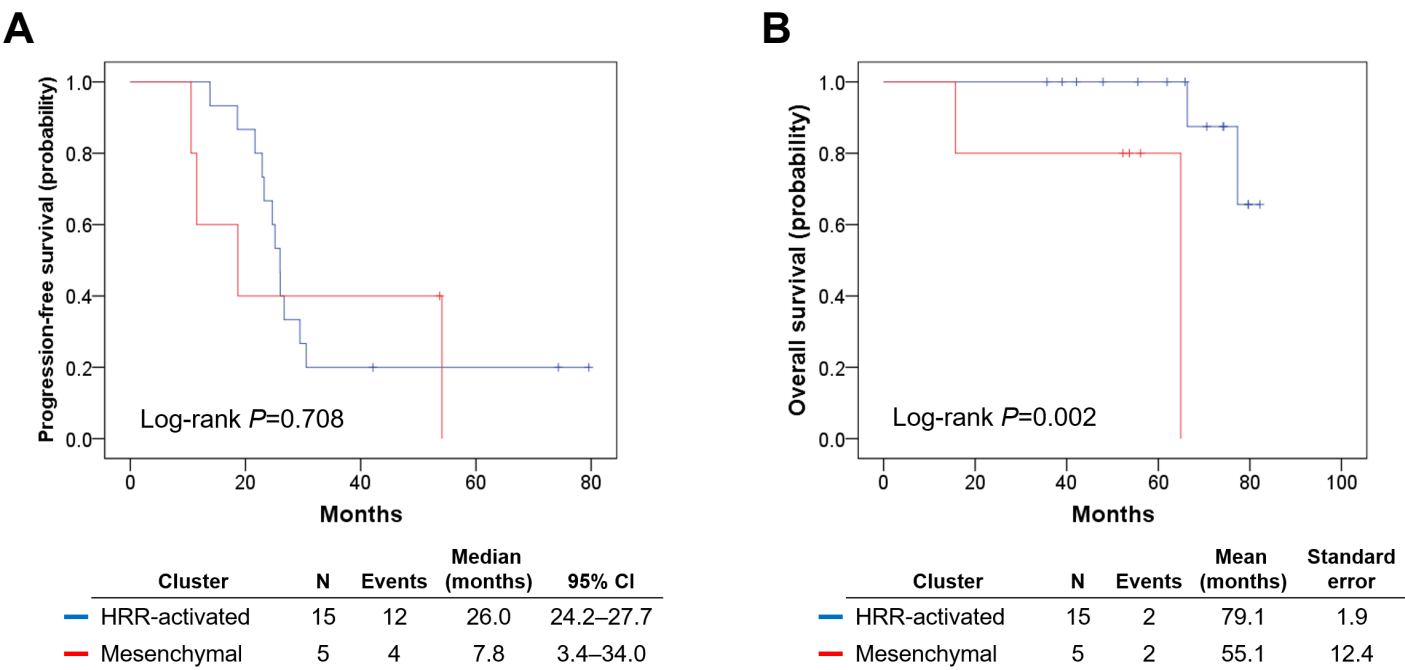

Supplement: Supplementary file 1 [file genes-12-01103-s001.zip › Figure S10.pdf]

Fig. S11

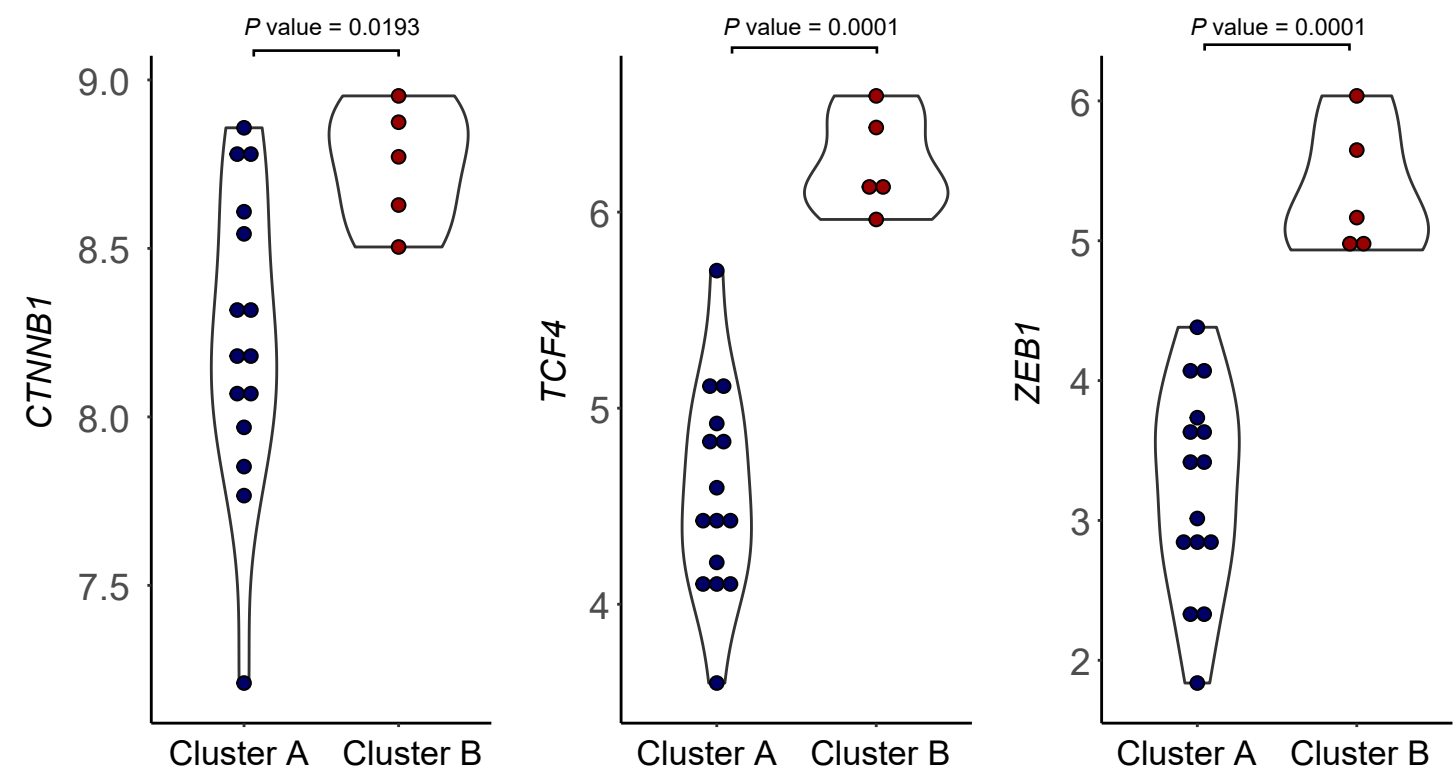

Supplement: Supplementary file 1 [file genes-12-01103-s001.zip › Figure S11.pdf]

Fig. S2

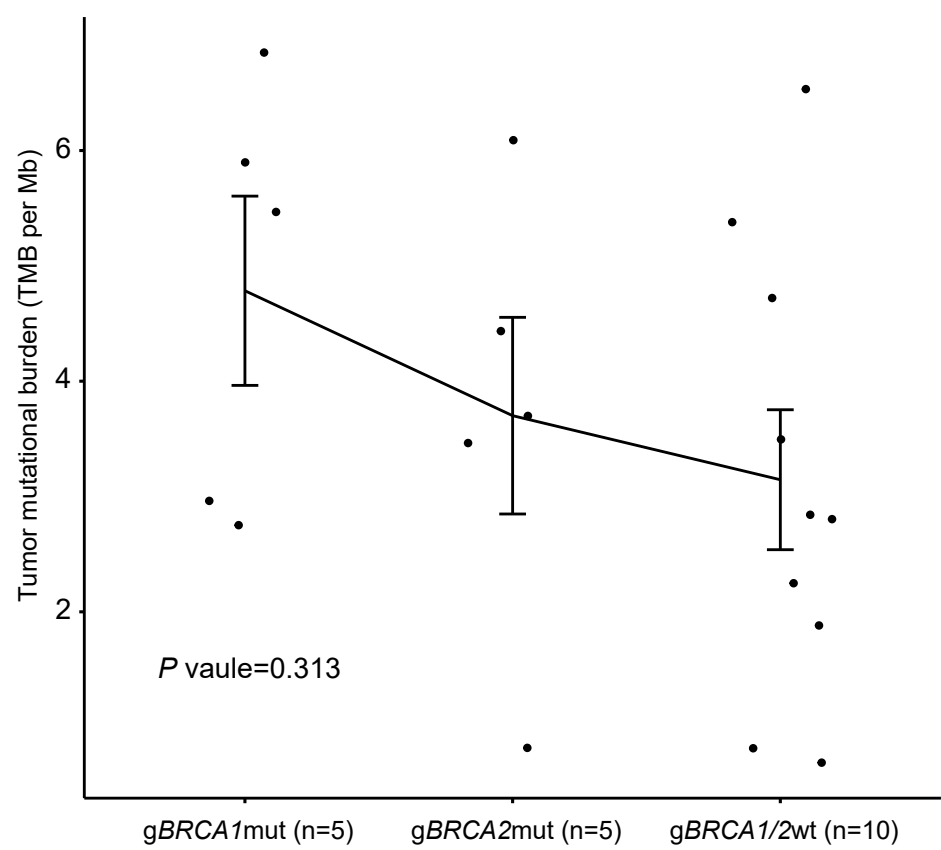

Supplement: Supplementary file 1 [file genes-12-01103-s001.zip › Figure S2.pdf]

Fig. S3

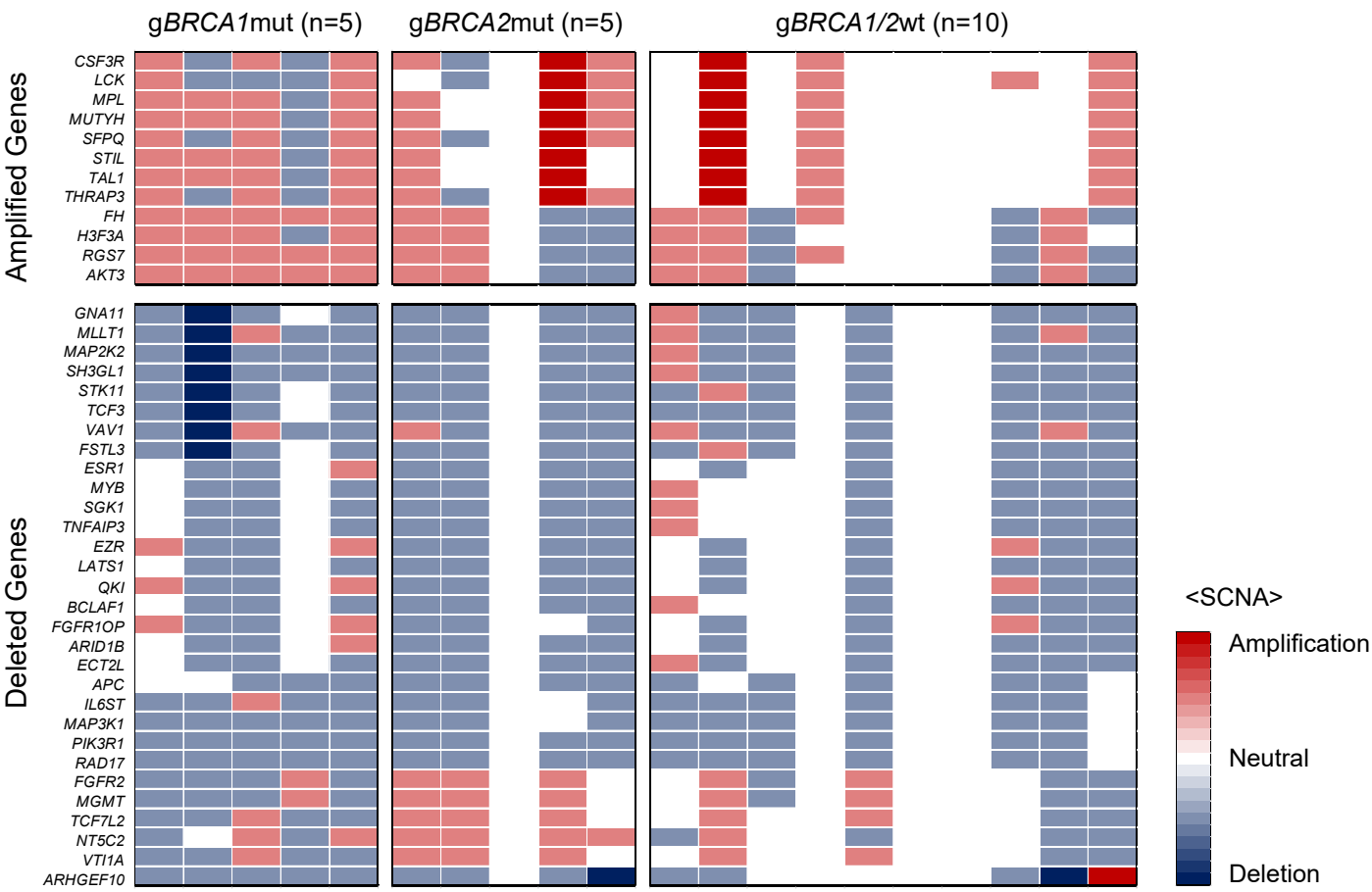

Supplement: Supplementary file 1 [file genes-12-01103-s001.zip › Figure S3.pdf]

Fig. S4

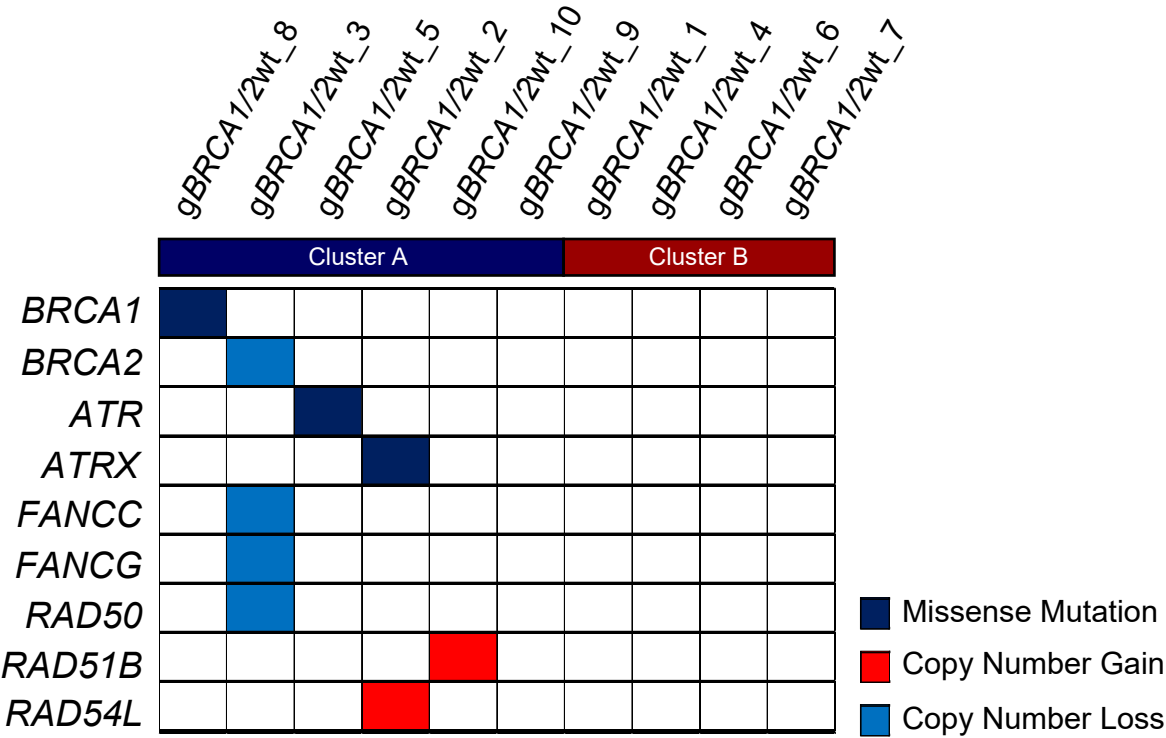

Supplement: Supplementary file 1 [file genes-12-01103-s001.zip › Figure S4.pdf]

Fig. S5

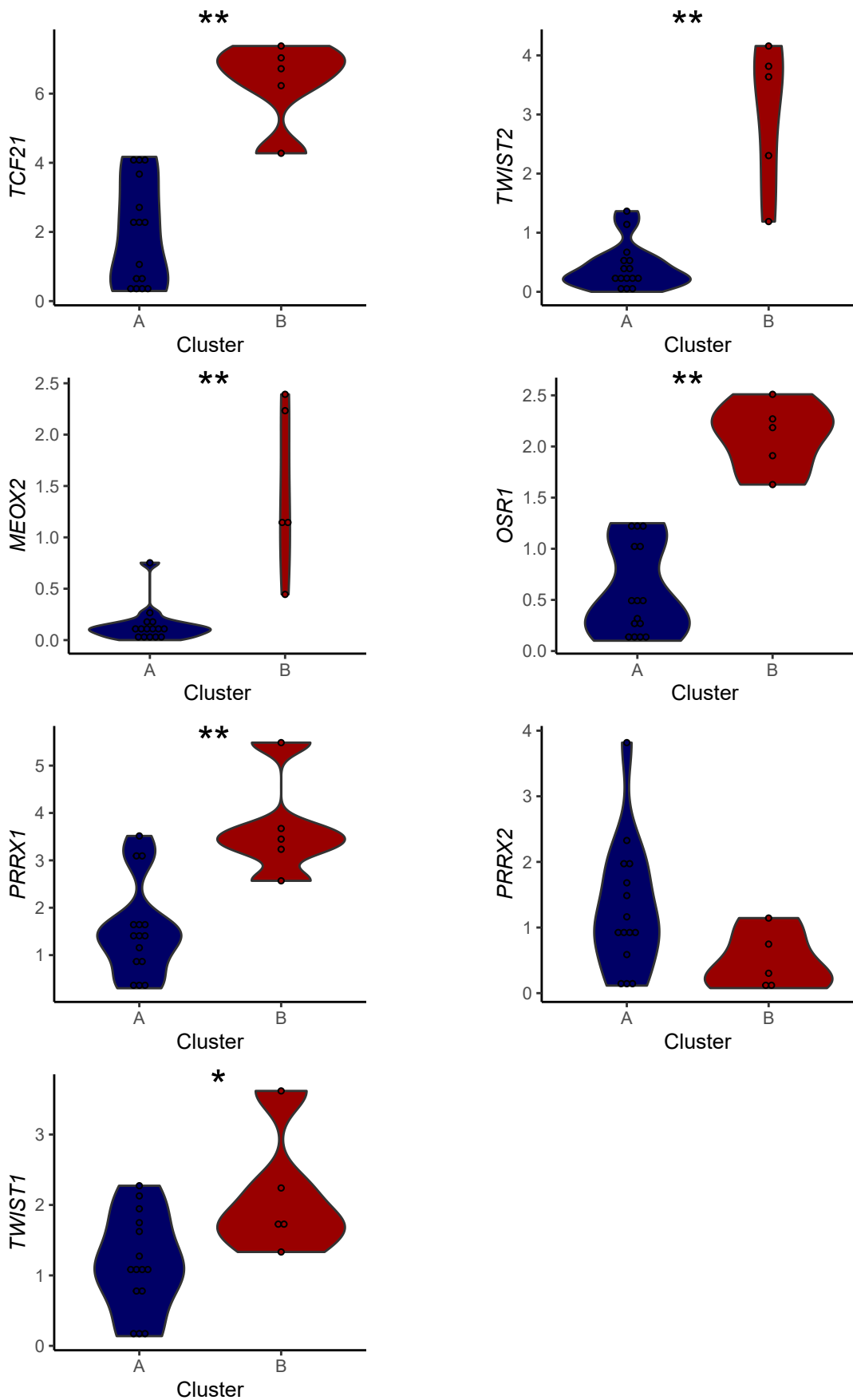

Supplement: Supplementary file 1 [file genes-12-01103-s001.zip › Figure S5.pdf]

Fig. S6

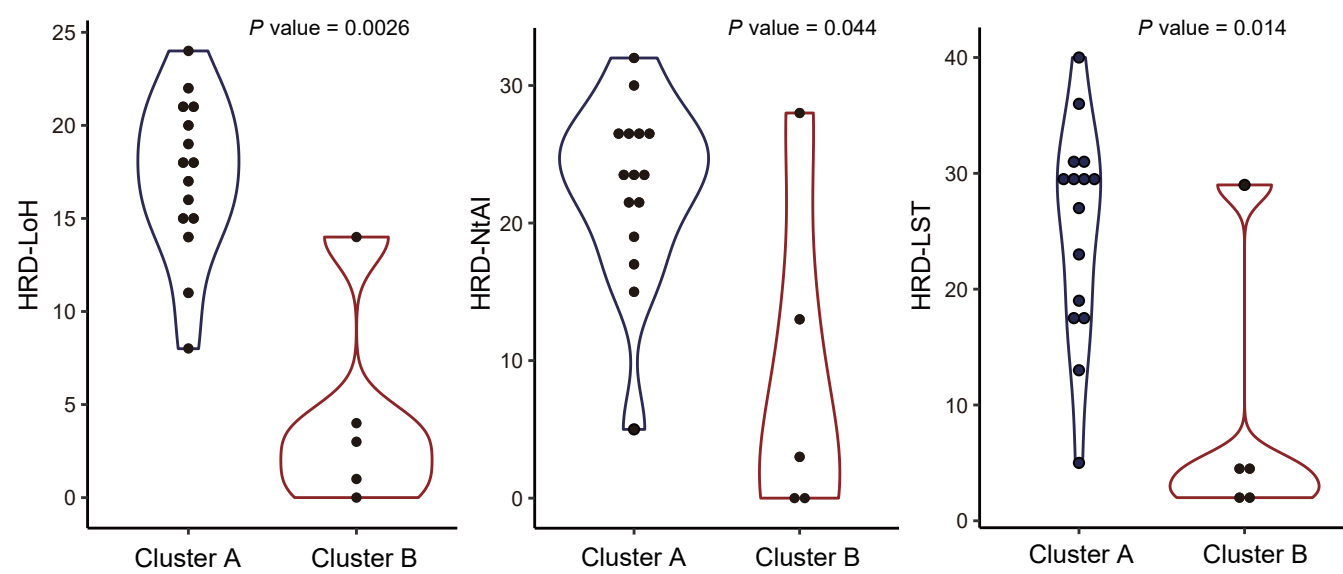

Supplement: Supplementary file 1 [file genes-12-01103-s001.zip › Figure S6.pdf]

Fig. S7

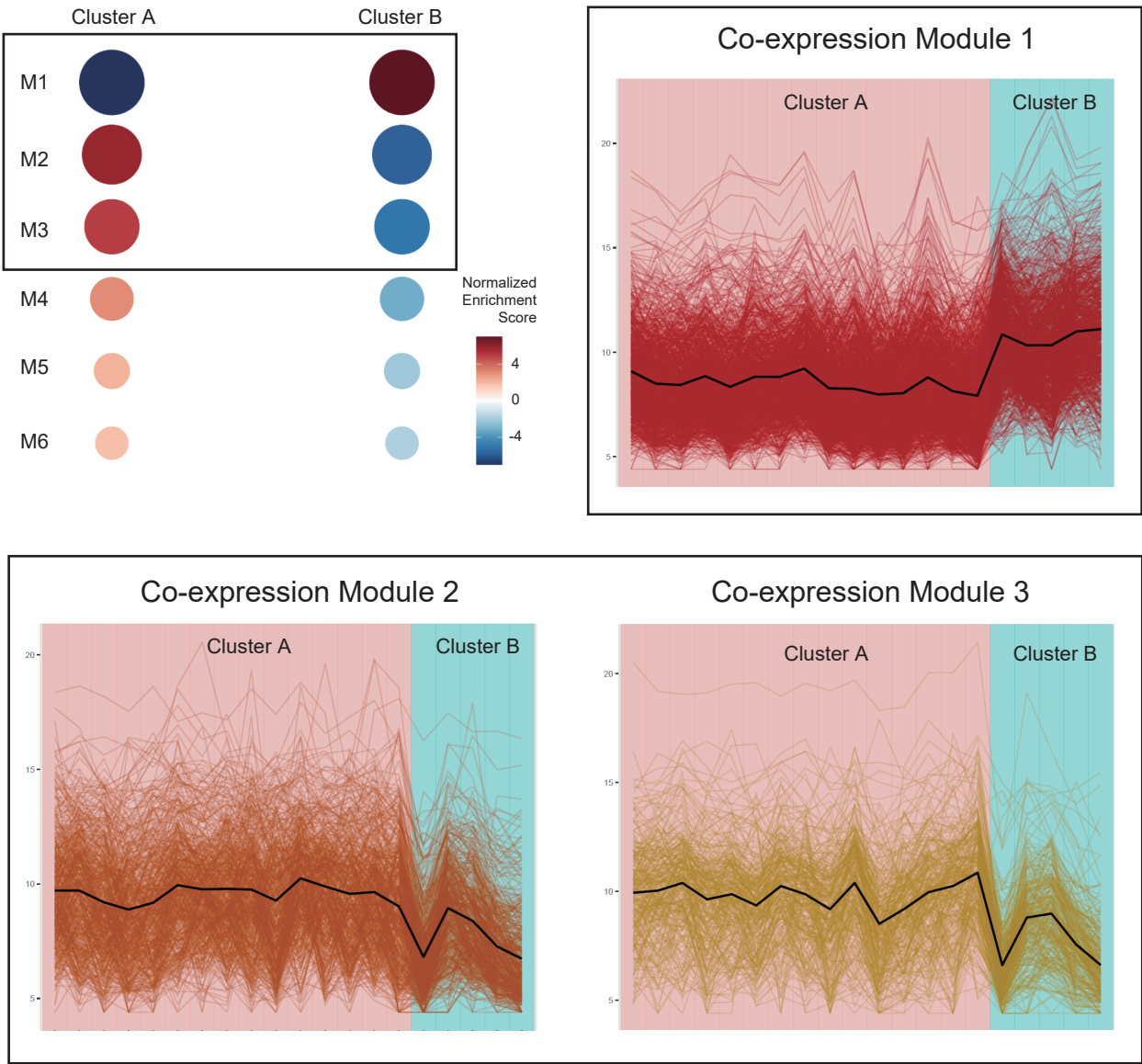

Supplement: Supplementary file 1 [file genes-12-01103-s001.zip › Figure S7.pdf]

Fig. S8

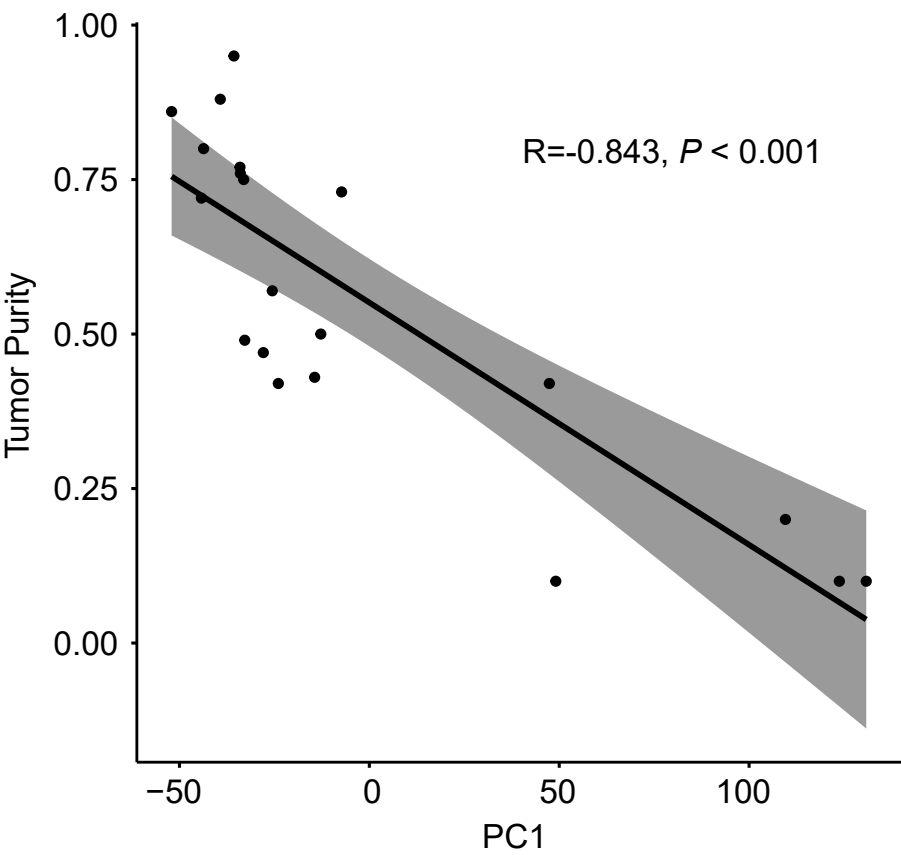

Supplement: Supplementary file 1 [file genes-12-01103-s001.zip › Figure S8.pdf]

**Fig. S9**

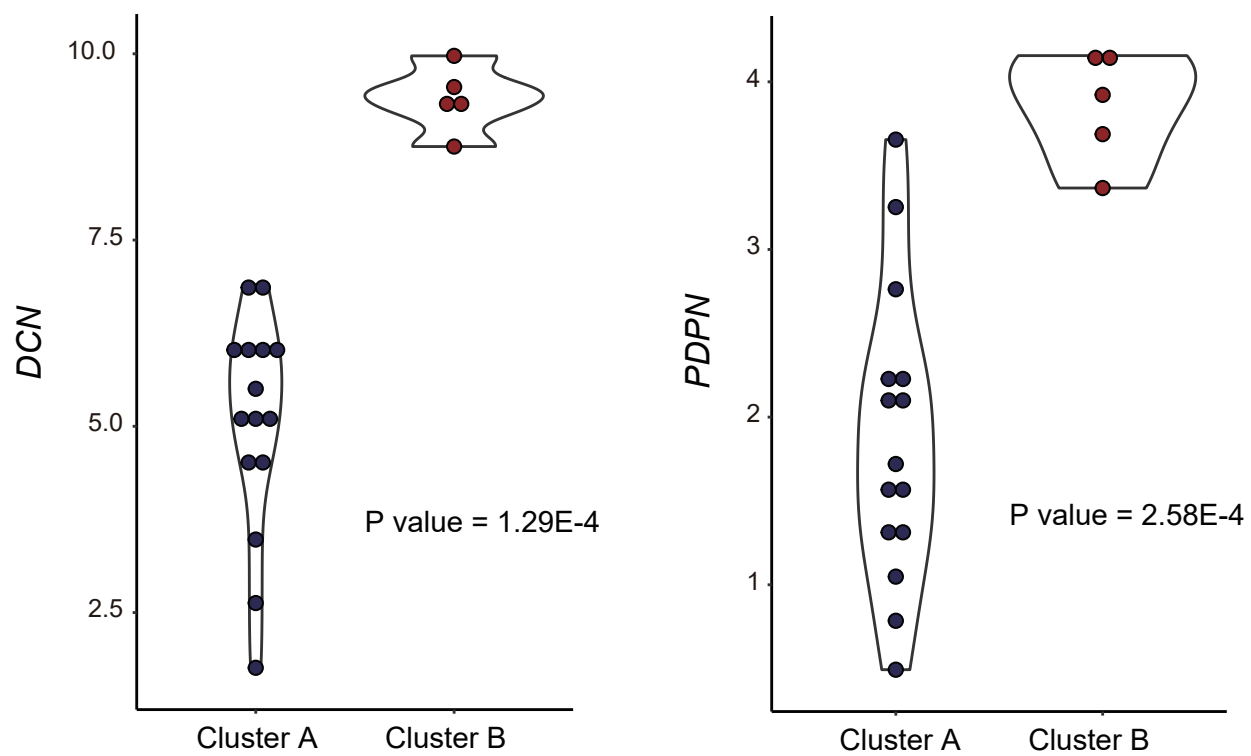

Supplement: Supplementary file 1 [file genes-12-01103-s001.zip › Figure S9.pdf]
